# Structure–activity studies in the development of a hydrazone based inhibitor of adipose-triglyceride lipase (ATGL)

**DOI:** 10.1016/j.bmc.2015.02.051

**Published:** 2015-06-15

**Authors:** Nicole Mayer, Martina Schweiger, Michaela-Christina Melcher, Christian Fledelius, Rudolf Zechner, Robert Zimmermann, Rolf Breinbauer

**Affiliations:** a Institute of Organic Chemistry, Graz University of Technology, Stremayrgasse 9, A-8010 Graz, Austria; b Institute of Molecular Biosciences, University of Graz, Heinrichstraße 31/II, A-8010 Graz, Austria; c Novo Nordisk A/S, Novo Nordisk Park, DK-2760 Måløv, Denmark

**Keywords:** Adipose triglyceride lipase, Hydrazones, Inhibitor, Lipid metabolism, Triglycerides

## Abstract

Adipose triglyceride lipase (ATGL) catalyzes the degradation of cellular triacylglycerol stores and strongly determines the concentration of circulating fatty acids (FAs). High serum FA levels are causally linked to the development of insulin resistance and impaired glucose tolerance, which eventually progresses to overt type 2 diabetes. ATGL-specific inhibitors could be used to lower circulating FAs, which can counteract the development of insulin resistance. In this article, we report about structure–activity relationship (SAR) studies of small molecule inhibitors of ATGL based on a hydrazone chemotype. The SAR indicated that the binding pocket of ATGL requests rather linear compounds without bulky substituents. The best inhibitor showed an IC_50_ = 10 μM in an assay with COS7-cell lysate overexpressing murine ATGL.

## Graphical abstract

**Figure:**
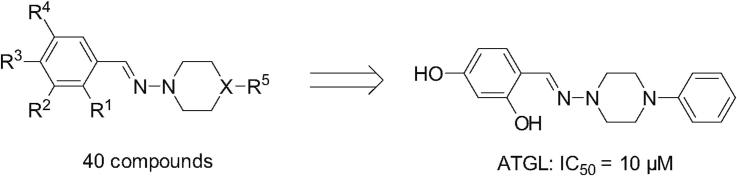

## 1 Introduction

During the last decades increasing evidence has emerged that fatty acid (FA) metabolism is closely linked to the development of metabolic disorders. Increased circulating FAs, as observed in obesity, can cause FA overload of non-adipose tissues resulting in ectopic triglyceride (TG) accumulation which is associated with impaired metabolic functions of these tissues, insulin resistance, and inflammation.^1^ Thus, decreasing plasma FA levels represents an apparent strategy to counteract the development of metabolic disease.

Plasma FA levels are strongly determined by lipases catalyzing the degradation of TG stores in adipose tissue. Efficient FA mobilization requires the activity of two lipases: Adipose triglyceride lipase (ATGL) and hormone-sensitive lipase (HSL). ATGL releases the first FA from the TG molecule and generates diacylglycerol (DG).^2^ Hormone-sensitive lipase (HSL) is the rate-limiting enzyme for the hydrolysis of DG,^3^ but is also capable of hydrolyzing TG and monoglycerides.^4^, ^5^ Since ATGL performs the first and rate-limiting step in lipolysis, inhibition of this enzyme is a powerful way to reduce FA availability. Accordingly, published data demonstrate that ATGL-deficient mice exhibit low plasma FA levels, improved glucose tolerance and insulin sensitivity, and are resistant to high-fat diet-induced insulin resistance.^6^, ^7^, ^8^, ^9^ Furthermore, pharmacological inhibition of ATGL reduces circulating FAs and triglyceride levels.^10^

It is important to note that defective ATGL function is associated with systemic TG accumulation in humans and rodents.^6^ Humans with defective ATGL function develop cardiomyopathy at the age of ∼30 years due to massive accumulation of TG in cardiomyocytes.^11^ Thus, it is reasonable to assume that also inhibitor-mediated ablation of ATGL activity can cause cardiac TG accumulation and myopathy. However, transient or partial inhibition of ATGL must not necessarily result in cardiac TG accumulation, since tissue TG stores can be rapidly mobilized to generate FA for energy conversion or as building blocks for complex lipids. Furthermore, small molecule inhibitors often show specific tissue distribution patterns which avoid their accumulation in cardiomyocytes, as recently demonstrated for the ATGL inhibitor Atglistatin.^10^

**Figure 1:**
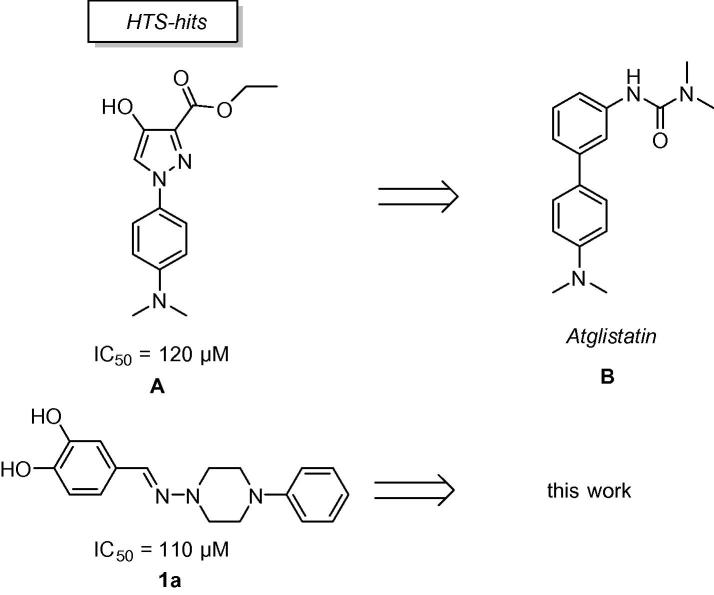
Hit compounds which served for the development of tool compounds against ATGL.

The significant biological relevance of ATGL makes it desirable to identify inhibitors of ATGL as tool compounds to study its biological role and validate ATGL as a potential drug target. However, at present no 3D-structure of ATGL is available and any effort to identify inhibitors of ATGL has to rely on the traditional approach of synthesizing and testing compounds. Recently, we have described a first inhibitor of murine ATGL, Atglistatin (**B**),^10^ which has been developed from a biaryl hit compound **A** resulting from a high-throughput screening (HTS) campaign originally aimed to inhibit HSL. In parallel to that work we have also followed a second hit compound **1a** based on a hydrazone chemotype, which had slightly better initial activity against ATGL (IC_50_ = 110 μM). Here we report about our efforts to develop **1a** as a tool compound and discuss the structure–activity data gained from this hydrazone chemotype (Fig. 1).

## 2 Results and discussion

### 2.1 Chemistry

Lead structure **1a** resulting from the HTS can be characterized by a hydrophobic side chain represented by the phenylpiperazine moiety connected with a catechol moiety via a hydrazone linkage. While we were aware about the problem associated with the intrinsic toxicity associated with hydrazones, we found the ready accessibility and synthetic flexibility of hydrazones attractive to gain fast insight into the structure–activity relationship of this inhibitor chemotype and hoped to be able to replace the hydrazone later by a more innocent functional group.^12^

**Scheme 1:**
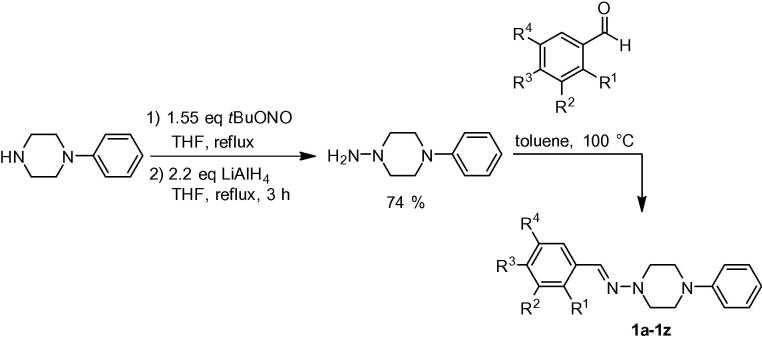
Synthesis of test compounds via hydrazone formation with aliphatic and arylic aldehydes.

**Scheme 2:**
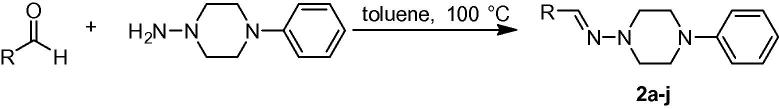
Synthesis of test compounds via hydrazone formation with aliphatic and arylic aldehydes.

In the first stage of our optimization studies we synthesized a set of compounds **1b**–**1z**, in which we varied the catechol substituent of hit compound **1a** by differently substituted aryl and aliphatic aldehydes in the hydrazone forming process (Scheme 1). Starting from commercial *N*-phenylpiperazine nitrosation with *tert*-butylnitrite^13^ followed by reduction with LiAlH_4_^14^ delivered the desired 4-phenylpiperazin-1-amine in 74% yield. This central nucleophile was then reacted with 26 differently substituted benzaldehydes in toluene at 100 °C producing the hydrazones **1a**–**1z** in 44–99% yield (Table 1).^15^ Similarly, another set of ten aliphatic and aromatic aldehydes was reacted with the same hydrazine under similar conditions to produce the hydrazones **2a**–**2j** in 82–99% yield (Scheme 2, Table 2).

**Table 1:** Screening data of hydrazones **1a**–**z** produced via Scheme 1

Entry	Compound	R1	R2	R3	R4	Yield (%)	IC_50_ (%)	*I*_200_ (%)
1	**1a**	–H	–OH	–OH	–H	92	110	69
2	**1b**	–H	–OMe	–OMe	–H	99	100	65
3	**1c**	–H	–OH	–OMe	–H	99	80	56
4	**1d**	–H	–OMe	–OH	–H	68	60	82
5	**1e**	–H	–OH	–H	–H	50	70	71
6	**1f**	–OH	–H	–H	–H	99	75	64
7	**1g**	–H	–OH	–H	–H	99	50	85
8	**1h**	–OH	–H	–OH	–H	99	10	94
9	**1i**	–H	–O*n*Pr	–H	–H	71	>200	14
10	**1j**	–H	–OPh	–H	–H	94	>200	24
11	**1k**	–OMe	–H	–OMe	–OMe	83	>200	43
12	**1l**	–H	–OMe	–OMe	–OMe	44	>200	41
13	**1m**	–H	–F	–F	–H	90	75	67
14	**1n**	–H	–Cl	–Cl	–H	90	>200	20
15	**1o**	–Cl	–H	–Cl	–H	95	>200	42
16	**1p**	–NO_2_	–H	–H	–H	97	180	60
17	**1q**	–Br	–H	–H	–H	89	>150	73
18	**1r**	–CN	–H	–H	–H	99	120	60
19	**1s**	–H	–H	–CN	–H	92	50	76
20	**1t**	–H	–H	–CH_3_	–H	99	>200	22
21	**1u**	–H	–H	–NMe_2_	–H	61	>200	5
22	**1v**	–H	–H	–NHAc	–H	96	>200	14
23	**1w**	–H	–H	–N(Me)(CH_2_)_2_OH	–H	84	>200	5
24	**1x**	–H	–OCH_2_O–	–OCH_2_O–	–H	87	>200	64
25	**1y**	–H	–O(CH_2_)_2_O–	–O(CH_2_)_2_O–	–H	95	>200	27
26	**1z**	–H	–NHCONH–	–NHCONH–	–H	99	>200	16

**Table 2:** Screening data of hydrazones **2a**–**j** produced via Scheme 2

Entry	Compound	R	Yield (%)	IC_50_ (%)	*I*_200_ (%)
1	**2a**		94	>200	12
2	**2b**		99	>200	12
3	**2c**		98	200	49
4	**2d**		94	130	58
5	**2e**		87	>200	16
6	**2f**		99	>200	46
7	**2g**		99	140	60
8	**2h**		82	>200	<1
9	**2i**		83	>200	8
10	**2j**		98	200	49

**Scheme 3:**
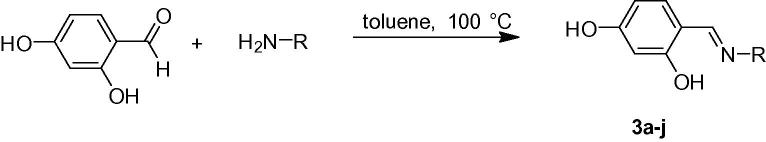
Synthetic access to second set of test compounds by variation of the piperazine moiety.

In the second stage of our studies we put our focus on the piperazine function by exchanging on the one hand the *N*-phenyl substituent at the piperazine by other *N*-aryl and *N*-alkyl substituents (compounds **3a**–**3g**, Table 3) and on the other hand by replacing the piperazine with a piperidine or homopiperazine ring (compounds **3h**–**3j**, Table 3). The proven substitution pattern from the hit compound representing the 2,4-dihydroxyphenylic system was kept constant in the aldehyde reactant in these studies (Scheme 3).

**Table 3:** Screening data of hydrazones **3a**–**j** produced via Scheme 3

Entry	Compound	R	Yield (%)	IC_50_ (%)	*I*_200_ (%)
1	**3a**		74	>200	19
2	**3b**		25	50	78
3	**3c**		58	>200	44
4	**3d**		95	>200	48
5	**3e**		95	>200	14
6	**3f**		66	>200	25
7	**3g**		76	>200	36
8	**3h**		57	40	81
9	**3i**		95	>200	26
10	**3j**		97	>200	87

**Scheme 4:**
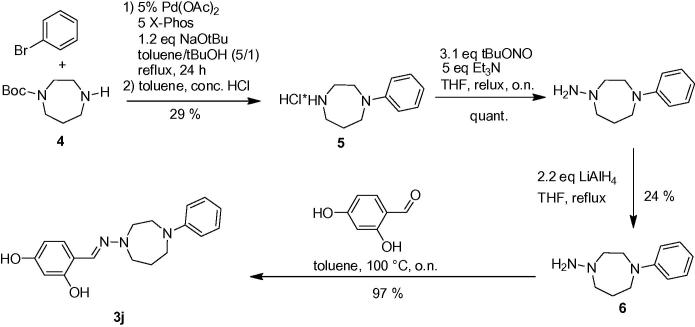
Synthetic route to homopiperazine compound **3j**.

The synthesis of the homopiperazine compound **3j** started with a Pd(0)-catalyzed Buchwald–Hartwig-arylation of Boc-protected homopiperazine (**4**) with bromobenzene using Pd(OAc)_2_/X-Phos with NaO*t*Bu as base,^16^ which after deprotection with HCl delivered the homopiperazine as its hydrochloride salt **5** in moderate 29% yield (Scheme 4). Nitrosylation of **5** and reduction with LiAlH_4_^14^ produced hydrazine **6** in 24% yield, which could then smoothly react with 2,4-dihydroxybenzaldehyde to the desired condensation product **3j** in 97% yield.

**Scheme 5:**
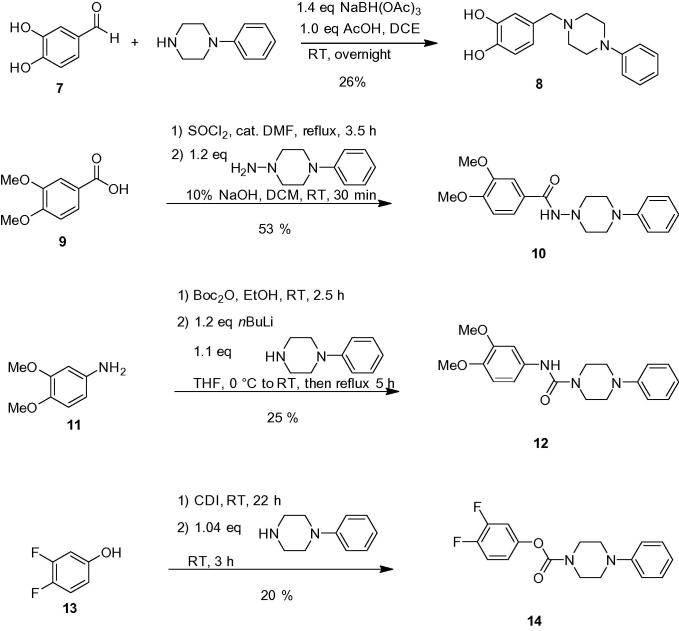
Synthesis of test compounds with hydrazone bioisosteres.

In the final stage of our structure activity studies we attempted to replace the hydrazone linkage with other linking units (Scheme 5). The more stable, but by one atom shorter, test compound **8** was prepared by reductive amination of 3,4-dihydroxybenzaldehyde (**7**) and *N*-phenylpiperidine with NaBH(OAc)_3_.^17^ For reasons of synthetic accessibility the 2,4-dihydroxyphenylic system was replaced in the synthesis of the following test compounds by other successful aldehyde fragments from the first stage of optimization, which did not have the reactive phenolic OH groups and were replaced by MeO and F substituents, which would also be less prone to phase 2 metabolism.^12^ The hydrazide analog **10** was synthesized via reaction of the acid chloride prepared from **9** with 4-phenylpiperazin-1-amine in 53% overall yield. For the synthesis of urea analog **12**, aniline **11** was first converted into the Boc-carbamate, which was then reacted with *N*-lithiated phenylpiperazine delivering **12** in 25% overall yield.^18^ Finally, urethane **14** was prepared by following a strategy,^19^ in which phenol **13** is first activated with 1,1′-carbonyldiimidazole (CDI) to an aryloxycarbonylimidazol and then reacted with 1-phenylpiperazine to produce the desired carbamate in 20% overall yield.

### 2.2 Biological activity

All synthesized test compounds were screened for their efficiency to inhibit ATGL activity in vitro in an assay using radiolabelled triolein as substrate.^20^ As ATGL cannot be purified, the assays were performed with COS7-cell lysates from cells overexpressing recombinant murine ATGL. For the determination of the IC_50_-value inhibitors were administered with increasing concentrations to the enzyme and its substrate. To determine the ATGL-inhibition efficiency at 200 μM (*I*_200_), 200 μM inhibitor was administered to the enzyme and its substrate. The results of the first set of test compounds **1a**–**1z** are summarized in Table 1. The IC_50_-values and the inhibition of the enzyme using an inhibitor concentration of 200 μM are listed. Starting from the hit compound **1a** from the HTS (entry 1) the influence of the HO substituents on the biological activity was investigated. As catechols are known to be oxidation sensitive and to be metabolized very fast in organisms, we varied the amount and position of HO substituents (**1e**–**1h**) and partly replaced them by methoxy groups (**1b**–**1d**). The activity increased in comparison to **1a** if one of the HO groups was methylated (entries 3 and 4). Even more pronounced was the activity gain if only a single HO substituent was present at the aldehyde building block (entries 5–7). The highest activity was observed for compound **1h** with its 2,4-dihydroxyphenyl moiety (IC_50_ = 10 μM, entry 8). If the HO substituents in the hit compound **1a** were substituted by fluorines the activity was slightly improved (IC_50_ = 75 μM, **1m**, entry 13), whereas the corresponding Cl-analog **1n** was inactive (entry 14) as also its structural isomer (entry 15). The comparison of *ortho*-hydroxylated compound **1f** with the analogous substrates **1p**–**1r** showed that –NO_2_, –Br or –CN substitution led to less active compounds (entries 16–18). From the data of the first screening it also became apparent, that bulky substituents or multiple substitution leading to sterically demanding compounds resulted in inactive compounds (entries 9–12 and 20–26). Interestingly, the rather linear 4-cyanosubstituted compound **1s** (IC_50_ = 50 μM, entry 19) had encouraging activity, while the bulkier 4-methylsubstituted analog **1t** (entry 20) was inactive, providing further evidence for a rather small and linear binding pocket of ATGL.

In the second set of screening compounds, the phenyl group of the aldehyde was replaced by alkyl, aryl and hetaryl groups (Table 2). From this set of compounds only the 1-cyclohexen-1-yl compound **2d** (entry 4) and 4-pyridyl compound **2g** (entry 7) showed moderate activity, while all other compounds were more or less inactive, corroborating the results from Table 1 that structures bulkier than the phenyl ring in hit compound **1a** seem to fit less well into the binding pocket.

In the next stage of our optimization studies we kept the best scoring fragment 2,4-dihydroxyphenyl (identified above from the screening of compounds in Table 1, Table 2) and varied the *N*-phenylpiperazine group. The results in Table 3 immediately suggest that any variation which leads to increased steric bulk by offering additional substituents or non-sp^2^-elements (entries 3–7, 9 and 10) were more or less inactive. Only the rather slim 4-fluorophenylpiperazine substrate **3b** (IC_50_ = 50 μM, entry 2) and the phenylpiperidino compound **3h** (IC_50_ = 40 μM, entry 8) resulted in active compounds, although the biological activity was lower in comparison to parent structure **1h**. The data direct to the following insights regarding the SAR: the space in the binding pocket seems to be limited as by introduction of nonplanarity in the case of cyclohexyl and cyclopentyl ring systems the activity gets lost and the hydrophobic π–π-interaction of an aromatic ring seems to be favorable.

As the hydrazone moiety is regarded as a problematic molecular group due to its metabolic instability resulting in toxic side products,^12^ we prepared compound **8** which can be regarded as a variant of **1a** missing one nitrogen atom (Scheme 5). However, this compound turned out to be biologically inactive (entry 1, Table 4). Although hydrazide **10** is a structurally very close analog to the active hydrazone **1b** (Table 1) the hydrazone/hydrazide switch resulted in an inactive compound (entry 2, Table 4). An inverso switch of the amido group of **10** led to urea compound **12**, which again showed no biological activity against ATGL (entry 3, Table 4). However, when introducing the carbamate moiety group in **14**, which is a structural analog of **1m** (entry 13, Table 1) an active but less potent compound (IC_50_ = 110 μM, entry 4, Table 4) could be generated, which might give rise to a covalent binding inhibitor based on the intrinsic reactivity of phenolcarbamates. Taken together these efforts have shown that the hydrazone moiety is an essential element of the biological activity of the hit compound **1a** and could not be replaced by the most obvious structural isosteres. In order to further characterize our hit compound **1a** and best inhibitor **1h**, these compounds were tested for ATGL- and HSL-selectivity by the determination of lipolysis in WAT (white adipose tissue) organ cultures of wild-type, ATGL-ko, and HSL-ko mice. Although the assay revealed a dose dependent reduction of FA release, lipolysis was similarly decreased in ATGL and HSL deficient adipose tissue indicating that the compounds **1a** and **1h** show no selectivity between ATGL and HSL (Suppl. Fig. 1).

**Table 4:** Screening data of alternative test compounds produced via Scheme 5

Entry	Compound	IC_50_ (%)	*I*_200_ (%)
1	**8**	>200	20
2	**10**	>200	38
3	**12**	>200	40
4	**14**	110	57

Further studies revealed that compound **1h** does not inhibit monoglyceride lipase (MGL), or lipoprotein lipase (Suppl. Fig. 2), but was determined to be toxic (Tox 4 toxicity test, Suppl. Fig. 3) suggesting that the hydrazone moiety contributes to this outcome.

## 3 Conclusion

In summary, we could show that hydrazones resulting from the condensation of electron rich benzaldehydes with 4-phenylpiperazin-1-amine result in quite active inhibitors of ATGL. The best compound **1h** exhibits respectable biological activity (IC_50_ = 10 μM) considering that the ATGL assay is performed with cell lysate instead of purified protein. While **1h** due to its limited specificity towards ATGL and intrinsic toxicity will be less useful as molecular probe than Atglistatin (**B**), the SAR studies presented in this manuscript have provided more insight into the characteristics of the ligand binding pocket of ATGL for the first time. These studies indicate that the binding pocket of ATGL is rather sensitive to variations of the investigated chemotype and that bulky substituents are problematic, suggesting that the binding pocket is rather narrow and thin, which might suit an enzyme binding lipids quite well.^21^, ^22^

## 4 Experimental section

### 4.1 Materials and methods

Acetonitrile, DMF, ethanol and dimethoxyethane were purchased as absolute solvents from Acros Organics, Fisher Scientific and Sigma Aldrich. Toluene (Sigma Aldrich, 99.7%) was dried in an aluminum oxide column under inert conditions and stored in Schlenk bottle over 4A molecule sieves under argon atmosphere. Tetrahydrofuran was dried at reflux temperature under argon atmosphere over sodium until benzophenone indicated dryness by a deep blue color. 1,2-Dichloroethane was purchased from ACROS Organics as extra dry solvent (99.8%, AcroSeal®) and directly used in the reactions. All applied starting materials were commercially available from Alfa Aesar and Sigma Aldrich and were used as received. Silica gel chromatography was performed with Acros Organics silica gel 60 (35–70 μM). ^1^H and ^13^C NMR spectra were recorded on a Bruker AVANCE III 300 spectrometer (^1^H: 300.36 MHz; ^13^C: 75.53 MHz) and chemical shifts are referenced to residual protonated solvent signals as internal standard. Electron impact (EI, 70 eV) HRMS spectra were recorded on Waters GCT Premier equipped with direct insertion (DI) and GC (HP GC7890A). Melting points were determined with the apparatus ‘Mel-Temp®’ from Electrothermal with an integrated microscopical support.

### 4.2 General synthesis

#### 4.2.1 1-Nitroso-4-phenylpiperazine

A 100 mL Schlenk tube was charged with 1.00 g (940 μL, 6.17 mmol, 1.00 equiv) 1-phenyl piperazine, 50 mL THF and 1.15 mL (9.57 mmol, 1.55 equiv) *tert* butylnitrite. The mixture was refluxed overnight. GC–MS analysis indicated full conversion of the starting material. The orange solution was transferred to a one-neck round bottom flask and concentrated in vacuum at a temperature of 30 °C. The crude product was used in the next reaction without further purification. Yield: 813.0 mg (74%), light brown solid. *R_f_* (MeOH/DCM 1:1) 0.55. ^1^H NMR (300 MHz, MeOD): *δ* (ppm) = 7.25–7.20 (m, 2H, Ar-H), 6.96 (d, ^3^*J* = 7.8 Hz, 2H, Ar-H), 6.84 (t, ^3^*J* = 7.2 Hz, 1H, Ar-H), 3.22 (br s, 4H, 2CH_2_), 2.82 (br s, 4H, 2CH_2_). ^13^C NMR (75.5 MHz, MeOD): *δ* (ppm) = 152.3 (Cq), 130.1 (2CHAr), 121.2 (CHAr), 117.6 (2CHAr), 59.2 (2CH_2_), 50.1 (2CH_2_). Mp: 36–38 °C.

#### 4.2.2 4-Phenylpiperazin-1-amine

A 250 mL three-neck round bottom flask with dropping funnel and reflux condenser was dried under vacuum, filled with nitrogen and charged with 516 mg (13.6 mmol, 2.20 equiv) LiAlH_4_ and 25 mL absolute THF. The grey suspension was heated under reflux and a solution of 1.18 g (6.18 mmol, 1.00 equiv) 1-nitroso-4-phenylpiperazine in 10 mL absolute THF was added dropwise to the boiling suspension. After complete addition the suspension was heated under reflux for further 3 h. GC–MS analysis showed full conversion of the starting material. The suspension was hydrolyzed according to the n,n,3n-method^23^ (1 mL water, 1 mL 15% aqueous NaOH and 3 mL water per 1 g LiAlH_4_) upon which the color turned to yellow. The mixture was filtrated through a fritted funnel, the filter cake was washed with 10 mL THF and the filtrate was concentrated under reduced pressure. Final purification by silica gel filtration (MeOH) yielded the pure product. Yield: 813.0 mg (74%), light brown solid. *R_f_* (MeOH/DCM 1:1) 0.55. ^1^H NMR (300 MHz, MeOD): *δ* (ppm) = 7.25–7.20 (m, 2H, Ar-H), 6.96 (d, ^3^*J* = 7.8 Hz, 2H, Ar-H), 6.84 (t, ^3^*J* = 7.2 Hz, 1H, Ar-H), 3.22 (br s, 4H, 2CH_2_), 2.82 (br s, 4H, 2CH_2_). ^13^C NMR (75.5 MHz, MeOD): *δ* (ppm) = 152.3 (Cq), 130.1 (2CH_Ar_), 121.2 (CH_Ar_), 117.6 (2CH_Ar_), 59.2 (2CH_2_), 50.1 (2CH_2_). Mp: 36–38 °C.

#### 4.2.3 General procedure (GP-1)

An aluminum reaction block was placed on a hotplate stirrer. A brown 10 mL reaction vessel was charged consecutively with 1.00 equiv 4-phenylpiperazin-1-amine, toluene, 1.00 equiv aldehyde and a magnetic stirring bar. The vessel was crimped with a cap, placed in the preheated (100 °C) reaction block and stirred vigorously at 100 °C. GC–MS analysis indicated full conversion of the starting material. After cooling to rt the cap was removed, the reaction mixture was transferred into a one-neck round bottom flask and concentrated under reduced pressure to yield the product.

#### 4.2.4 (*E*)-4-((4-Phenylpiperazin-1-ylimino)methyl)benzene-1,2-diol (**1a**)

According to GP-1: 50.0 mg (282 μmol, 1.00 equiv) 4-phenylpiperazin-1-amine, 39.0 mg (282 μmol, 1.00 equiv) 3,4-dihydroxybenzaldehyde, 1.0 mL toluene, stirring for 2.5 h. temperature of 30 °C. Yield: 77.0 mg (92%), brown solid. ^1^H NMR (300 MHz, DMSO-*d*_6_): *δ* (ppm) = 7.58 (s, 1H, CH=N), 7.26–7.21 (m, 2H, Ar-H), 7.10 (d, ^4^*J* = 1.8 Hz, 1H, Ar-H), 7.00 (t, ^3^*J* = 8.1 Hz, 2H, Ar-H), 6.83–6.78 (m, 2H, Ar-H), 6.72 (d, ^3^*J* = 8.1 Hz, 1H, Ar-H), 3.30–3.28 (m, 4H, 2CH_2_), 3.17–3.14 (m, 4H, 2CH_2_). ^13^C NMR (75.5 MHz, DMSO-*d*_6_): *δ* (ppm) = 150.6 (Cq), 146.0 (Cq-OH), 145.3 (Cq-OH), 137.4 (CH=N), 128.9 (2CHAr), 127.7 (Cq), 119.1 (CHAr), 118.6 (CHAr), 115.8 (2CHAr), 115.3 (CHAr), 112.1 (CHAr), 51.0 (2CH_2_), 47.8 (2CH_2_). Mp: 180 °C.

#### 4.2.5 (*E*)-*N*-(3,4-Dimethoxybenzylidene)-4-phenylpiperazin-1-amine (**1b**)

According to GP-1: 10.0 mg (56.0 μmol, 1.20 equiv) 4-phenylpiperazin-1-amine, 7.80 mg (47.0 μmol, 1.00 equiv) 3,4-dimethoxybenzaldehyde, 0.5 mL toluene, stirring for 4 h. Yield: 15.3 mg (>99%), yellow-brown solid. ^1^H NMR (300 MHz, CDCl_3_): *δ* (ppm) = 7.62 (br s, 1H, CH=N), 7.36–7.27 (m, 3H, Ar-H), 7.05–6.98 (m, 3H, Ar-H), 6.93–6.84 (m, 2H, Ar-H), 3.94 (s, 3H, OCH_3_), 3.90 (s, 3H, OCH_3_), 3.40–3.38 (m, 4H, 2CH_2_), 3.34–3.32 (m, 4H, 2CH_2_). ^13^C NMR (75.5 MHz, CDCl_3_): *δ* (ppm) = 150.9 (2Cq-OCH_3_), 149.6 (Cq), 149.3 (Cq), 137.3 (CH=N), 129.2 (2CHAr), 120.4 (CHAr), 120.2 (CHAr), 116.5 (2CHAr), 110.7 (CHAr), 107.5 (CHAr), 55.9 (OCH_3_), 55.8 (OCH_3_), 51.4 (2CH_2_), 48.9 (2CH_2_). Mp: 153–154 °C.

#### 4.2.6 (*E*)-2-Methoxy-5-((4-phenylpiperazin-1-ylimino)methyl)phenol (**1c**)

According to GP-1: 10.0 mg (56.0 μmol, 1.10 equiv) 4-phenylpiperazin-1-amine, 7.80 mg (51 μmol, 1.00 equiv) 3-hydroxyanisaldehyde, 0.5 mL toluene, stirring for 4.5 h. Yield: 16.0 mg (>99%), yellow solid. ^1^H NMR (300 MHz, DMSO-*d*_6_): *δ* (ppm) = 9.06 (s, 1H, OH), 7.62 (s, 1H, CH=N), 7.26–7.21 (m, 2H, Ar-H), 7.12 (d, ^4^*J* = 1.5 Hz, 1H, Ar-H), 7.02–6.79 (m, 5H, Ar-H), 3.77 (s, 3H, OCH_3_), 3.31–3.29 (m, 4H, 2CH_2_), 3.20–3.18 (m, 4H, 2CH_2_). ^13^C NMR (75.5 MHz, DMSO-*d*_6_): *δ* (ppm) = 150.6 (Cq), 148.0 (Cq-OCH_3_), 146.5 (Cq-OH), 136.7 (CH=N), 129.2 (Cq), 128.9 (2CHAr), 119.1 (CHAr), 118.3 (CHAr), 115.8 (2CHAr), 111.8 (CHAr), 111.7 (CHAr) 55.5 (OCH_3_), 50.9 (2CH_2_), 47.8 (2CH_2_). Mp: 180 °C (dec.).

#### 4.2.7 (*E*)-2-Methoxy-4-((4-phenylpiperazin-1-ylimino)methyl)phenol (**1d**)

According to GP-1: 44.0 mg (249 μmol, 1.00 equiv) 4-phenylpiperazin-1-amine, 31.3 mg (205 μmol, 1.00 equiv) vanilline, 1.0 mL toluene, stirring overnight, recrystallization from 2.5 mL toluene. Yield: 48.0 mg (68%), brown oil. ^1^H NMR (300 MHz, DMSO-*d*_6_): *δ* (ppm) = 9.18 (s, 1H, OH), 7.66 (s, 1H, CH=N), 7.26–7.16 (m, 3H, Ar-H), 7.02–6.97 (m, 3H, Ar-H), 6.83–6.75 (m, 2H, Ar-H), 3.78 (s, 3H, OCH_3_), 3.31–3.19 (m, 8H, 4CH_2_). ^13^C NMR (75.5 MHz, DMSO-*d*_6_): *δ* (ppm) = 150.6 (Cq), 147.7 (Cq-OCH_3_), 147.0 (Cq-OH), 137.2 (CH=N), 128.9 (2CHAr), 128.1 (Cq), 127.7 (CHAr), 119.9 (CHAr), 115.8 (2CHAr), 115.3 (CHAr), 108.6 (CHAr), 55.4 (OCH_3_), 51.0 (2CH_2_), 47.8 (2CH_2_).

#### 4.2.8 (*E*)-3-((4-Phenylpiperazin-1-ylimino)methyl)phenol (**1e**)

According to GP-1: 40.0 mg (226 μmol, 1.00 equiv) 4-phenylpiperazin-1-amine, 27.6 mg (226 μmol, 1.00 equiv) 3-hydroxybenzaldehyde, 1.0 mL toluene, stirring overnight. Yield: 32.0 mg (50%), yellow solid. ^1^H NMR (300 MHz, DMSO-*d*_6_): *δ* (ppm) = 9.41 (s, 1H, OH), 7.64 (s, 1H, CH=N), 7.27–7.21 (m, 2H, Ar-H), 7.15 (t, ^3^*J* = 7.8 Hz, 1H, Ar-H), 7.06–6.99 (m, 4H, Ar-H), 6.81 (t, ^3^*J* = 7.2 Hz, 1H, Ar-H), 6.68 (dd, ^4^*J* = 1.5 Hz, ^3^*J* = 7.8 Hz, 1H, Ar-H), 3.32–3.30 (m, 4H, 2CH_2_), 3.24–3.23 (m, 4H, 2CH_2_). ^13^C NMR (75.5 MHz, DMSO-*d*_6_): *δ* (ppm) = 157.4 (Cq-OH), 150.6 (Cq), 137.4 (Cq), 136.1 (CH=N), 129.3 (CHAr), 128.9 (2CHAr), 119.1 (CHAr), 117.3 (CHAr), 115.8 (2CHAr), 115.2 (CHAr), 111.7 (CHAr) 50.6 (2CH_2_), 47.7 (2CH_2_). Mp: 190 °C (dec.).

#### 4.2.9 (*E*)-2-((4-Phenylpiperazin-1-ylimino)methyl)phenol (**1f**)

According to GP-1: 40.0 mg (226 μmol, 1.00 equiv) 4-phenylpiperazin-1-amine, 27.6 mg (24.0 μL, 226 μmol, 1.00 equiv) 3-hydroxybenzaldehyde, 1.0 mL toluene, stirring overnight. Addition of 14.0 mg (79.0 μmol, 0.35 equiv) 4-phenylpiperazin-1-amine after 15 h to reach full conversion of the starting material after further 6 h stirring. Yield: 84.0 mg (99%), yellow solid. ^1^H NMR (300 MHz, CDCl_3_): *δ* (ppm) = 11.54 (s, 1H, OH), 7.80 (s, 1H, CH=N), 7.35–7.16 (m, 4H, Ar-H), 7.06–6.88 (m, 5H, Ar-H), 3.41–3.38 (m, 8H, 4 CH_2_). ^13^C NMR (75.5 MHz, CDCl_3_): *δ* (ppm) = 157.7 (Cq-OH), 141.6 (CH=N), 129.8 (CHAr), 129.7 (2CHAr), 129.2 (2CHAr), 129.0 (Cq), 120.6 (Cq), 119.1 (CHAr), 118.9 (CHAr), 116.7 (CHAr), 116.6 (CHAr), 51.3 (2CH_2_), 48.7 (2CH_2_). Mp: 138–142 °C.

#### 4.2.10 (*E*)-4-((4-Phenylpiperazin-1-ylimino)methyl)phenol (**1g**)

According to GP-1: 40.0 mg (226 μmol, 1.00 equiv) 4-phenylpiperazin-1-amine, 27.6 mg (226 μmol, 1.00 equiv) 4-hydroxybenzaldehyde, 1.0 mL toluene, stirring overnight. Yield: 63.5 mg (>99%), beige solid. ^1^H NMR (300 MHz, DMSO-*d*_6_): *δ* (ppm) = 9.59 (s, 1H, OH), 7.66 (s, 1H, CH=N), 7.42 (d, ^3^*J* = 8.7 Hz, 2H, Ar-H), 7.25–7.20 (m, 2H, Ar-H), 7.00 (d, ^3^*J* = 8.1 Hz, 2H, Ar-H), 6.83–6.74 (m, 3H, Ar-H), 3.30–3.28 (m, 4H, 2CH_2_), 3.18–3.16 (m, 4H, 2CH_2_). ^13^C NMR (75.5 MHz, DMSO-*d*_6_): *δ* (ppm) = 157.6 (Cq-OH), 150.6 (Cq), 137.1 (CH=N), 128.9 (2CHAr), 127.3 (2CHAr), 127.2 (Cq), 119.1 (CHAr), 115.7 (2CHAr), 115.3 (2CHAr), 51.0 (2CH_2_), 47.8 (2CH_2_). Mp: 192 °C (dec.).

#### 4.2.11 (*E*)-4-(4-Phenylpiperazin-1-ylimino)methylbenzene-1,3-diol (**1h**)

According to GP-1: 30.0 mg (169 μmol, 1.00 equiv) 4-phenylpiperazin-1-amine, 23.4 mg (169 μmol, 1.00 equiv) 2,4-dihydroxybenzaldehyde), 1.0 mL toluene, stirring for 2 h. Yield: 51.0 mg (>99%), brown solid. *R_f_* (MeOH): 0.62. ^1^H NMR (300 MHz, DMSO-*d*_6_): *δ* (ppm) = 11.60 (s, 1H, OH), 9.72 (br s, 1H, OH), 7.95 (s, 1H, CH=N), 7.26–7.16 (m, 3H, Ar-H), 7.00 (d, ^3^*J* = 8.1 Hz, 2H, Ar-H), 6.81 (t, ^3^*J* = 7.2 Hz, 1H, Ar-H), 6.32 (dd, ^3^*J* = 8.1 Hz, ^4^*J* = 2.1 Hz, 1H, Ar-H), 6.25 (d, ^4^*J* = 2.1 Hz, 1H, Ar-H), 3.33–3.30 (m, 4H, 2CH_2_), 3.18–3.15 (m, 4H, 2CH_2_). ^13^C NMR (75.5 MHz, DMSO-*d*_6_): *δ* (ppm) = 159.0 (Cq-OH), 158.6 (Cq-OH), 150.5 (Cq), 142.3 (CH=N), 130.7 (CHAr), 128.9 (2CHAr), 119.2 (CHAr), 115.8 (2CHAr), 111.4 (Cq), 107.0 (CHAr), 102.4 (CHAr), 51.1 (2CH_2_), 47.5 (2CH_2_). Mp: 183–184 °C.

#### 4.2.12 (*E*)-4-Phenyl-*N*-(4-propoxybenzylidene)piperazin-1-amine (**1i**)

According to GP-1: 30.0 mg (169 μmol, 1.00 equiv) 4-phenylpiperazin-1-amine, 27.7 mg (27.0 μL, 169 μmol, 1.00 equiv) 4-propoxybenzaldehyde, 1.0 mL toluene, stirring overnight. Yield: 40.0 mg (71%), light yellow solid. ^1^H NMR (300 MHz, CDCl_3_): *δ* (ppm) = 7.65 (s, 1H, CH=N), 7.57 (d, ^3^*J* = 8.7 Hz, 2H, Ar-H), 7.33–7.28 (m, 2H, Ar-H), 6.99 (d, ^3^*J* = 8.1 Hz, 2H, Ar-H), 6.93–6.88 (m, 3H, Ar-H), 3.94 (t, ^3^*J* = 6.6 Hz, 2H, OCH_2_), 3.41–3.38 (m, 4H, 2CH_2_), 3.33–3.30 (m, 4H, 2CH_2_), 1.82 (sext, ^3^*J* = 6.9 Hz, ^3^*J* = 7.2 Hz, 2H, CH_2_), 1.05 (t, ^3^*J* = 7.2 Hz, 3H, CH_3_). ^13^C NMR (75.5 MHz, CDCl_3_): *δ* (ppm) = 159.6 (Cq-OCH_2_), 150.9 (Cq), 137.7 (CH=N), 129.2 (2CHAr), 128.6 (Cq), 127.6 (2CHAr), 120.2 (CHAr), 116.5 (2CHAr), 114.6 (2CHAr), 69.5 (OCH_2_), 51.5 (2CH_2_), 48.9 (2CH_2_), 22.6 (CH_2_), 10.5 (CH_3_). Mp: 184–185 °C.

#### 4.2.13 (*E*)-*N*-(4-Phenoxybenzylidene)-4-phenylpiperazin-1-amine (**1j**)

According to GP-1: 40.0 mg (226 μmol, 1.00 equiv) 4-phenylpiperazin-1-amine, 44.7 mg (226 μmol, 1.00 equiv) 4-phenoxybenzaldehyde, 1.0 mL toluene, stirring overnight. Yield: 76.0 mg (94%), yellow-brown solid. ^1^H NMR (300 MHz, CDCl_3_): *δ* (ppm) = 7.66 (s, 1H, CH=N), 7.61 (d, ^3^*J* = 8.7 Hz, 2H, Ar-H), 7.38–7.28 (m, 4H, Ar-H), 7.12 (t, ^3^*J* = 7.2 Hz, 1H, Ar-H), 7.02 (m, 6H, Ar-H), 6.92 (t, ^3^*J* = 7.2 Hz, 1H, Ar-H), 3.41–3.34 (m, 8H, 4 CH_2_). ^13^C NMR (75.5 MHz, CDCl_3_): *δ* (ppm) = 157.6 (Cq), 156.9 (Cq), 150.9 (Cq), 136.4 (CH=N), 131.2 (Cq), 129.8 (2CHAr), 129.2 (2CHAr), 127.7 (2CHAr), 123.4 (2CHAr), 120.3 (CHAr), 119.0 (2CHAr), 118.8 (2CHAr), 116.6 (CHAr), 51.3 (2CH_2_), 48.9 (2CH_2_). Mp: 161–164 °C. HRMS (EI^+^): *m*/*z*: calcd for C_23_H_23_ON_3_ [M]^+^: 357.1841; found 357.1844.

#### 4.2.14 (*E*)-4-Phenyl-*N*-(2,4,5-trimethoxybenzyliden)piperazin-1-amine (**1k**)

According to GP-1: 30.0 mg (169 μmol, 2.00 equiv) 4-phenylpiperazin-1-amine, 33.1 mg (169 μmol, 1.00 equiv) 2,4,5-trimethoxybenzaldehyde, 1.0 mL toluene, stirring overnight. Addition of 3.0 mg (15.0 μmol, 0.09 equiv) 4-phenylpiperazin-1-amine after 18 h to reach full conversion of the starting material after further stirring over a second night. Yield: 55.0 mg (83%), yellow-orange solid. ^1^H NMR (300 MHz, CDCl_3_): *δ* (ppm) = 8.00 (br s, 1H, CH=N), 7.46 (s, 1H, Ar-H), 7.34–7.27 (m, 3H, Ar-H), 6.99 (d, ^3^*J* = 7.8 Hz, 2H, Ar-H), 6.50 (s, 1H, arom.H), 3.91 (s, 3H, OCH_3_), 3.90 (s, 3H, OCH_3_), 3.85 (s, 3H, OCH_3_), 3.41–3.38 (m, 4H, 2CH_2_), 3.34–3.31 (m, 4H, 2CH_2_). ^13^C NMR (75.5 MHz, CDCl_3_): *δ* (ppm) = 152.1 (Cq-OCH_3_), 151.0 (Cq-OCH_3_), 150.3 (Cq-OCH_3_), 143.7 (CH=N), 129.1 (2CHAr), 128.2 (Cq), 120.1 (CHAr), 116.6 (Cq), 116.5 (2CHAr), 107.9 (CHAr), 97.3 (CHAr), 56.7 (OCH_3_), 56.2 (OCH_3_), 56.0 (OCH_3_), 51.5 (2CH_2_), 48.9 (2CH_2_). Mp: 153–156 °C.

#### 4.2.15 (*E*)-4-Phenyl-*N*-(3,4,5-trimethoxybenzylidene)piperazin-1-amine (**1l**)

According to GP-1: 30.0 mg (169 μmol, 2.00 equiv) 4-phenylpiperazin-1-amine, 33.1 mg (169 μmol, 1.00 equiv) 3,4,5-trimethoxybenzaldehyde, 1.0 mL toluene, stirring overnight. Addition of 2.00 mg (10.0 μmol, 0.06 equiv) 4-phenylpiperazin-1-amine after 18 h to reach full conversion of the starting material after further stirring over a second night. Yield: 28.0 mg (44%), yellow solid. ^1^H NMR (300 MHz, CDCl_3_): *δ* (ppm) = 7.93 (s, 1H, CH=N), 7.70–7.64 (m, 3H, Ar-H), 7.37 (d, ^3^*J* = 8.1 Hz, 2H, Ar-H), 7.33–7.28 (m, 2H, Ar-H), 4.28 (s, 6H, 2OCH_3_), 4.24 (s, 3H, OCH_3_), 3.78–3.71 (m, 8H, 4 CH_2_). ^13^C NMR (75.5 MHz, CDCl_3_): *δ* (ppm) = 153.4 (2Cq-OCH_3_), 150.9 (Cq-OCH_3_), 138.5 (Cq), 136.4 (CH=N), 131.7 (Cq), 129.2 (2CHAr), 120.3 (CHAr), 116.6 (2CHAr), 103.2 (CHAr), 60.9 (OCH_3_), 56.1 (2OCH_3_), 51.2 (2CH_2_), 48.9 (2CH_2_). Mp: 93–98 °C.

#### 4.2.16 (*E*)-*N*-(3,4-Difluorobenzylidene)-4-phenylpiperazin-1-amine (**1m**)

According to GP-1: 250 mg (1.41 mmol, 2.00 equiv) 4-phenylpiperazin-1-amine, 100 mg (78.0 μL, 706 μmol, 1.00 equiv) 3,4-difluorobenzaldehyde, 3.0 mL toluene, stirring overnight. Yield: 189.8 g (90%), yellow solid. ^1^H NMR (300 MHz, CDCl_3_): *δ* (ppm) = 7.52–7.43 (m, 2H, CH=N, Ar-H), 7.31–7.24 (m, 3H, Ar-H), 7.16–7.07 (m, 1H, Ar-H), 6.98 (d, ^3^*J* = 7.8 Hz, 2H, Ar-H), 6.90 (t, ^3^*J* = 7.2 Hz, 1H, Ar-H), 3.37–3.36–3.34 (m, 8H, 4CH_2_). ^13^C NMR (75.5 MHz, CDCl_3_): *δ* (ppm) = 152.4, 152.2 (^3^*J*_C–F_ = 16.5 Hz, Cq), 151.8, 149.1 (^1^*J*_C–F_ = 203.2 Hz, Cq-F), 150.8, 148.7 (^1^*J*_C–F_ = 161.2 Hz, Cq-F), 133.6 (CH=N), 133.5 (Cq), 129.2 (2CHAr), 122.5, 122.4, 122.4, 122.3 (^3^*J*_C–F_ = 6.2 Hz, ^4^*J*_C–F_ = 3.2 Hz, CHAr), 120.4 (CHAr), 117.3, 117.1 (^2^*J*_C–F_ = 17.6 Hz, CHAr), 116.6 (2CHAr), 114.2, 114.0 (^2^*J*_C–F_ = 18.2 Hz, CHAr), 51.0 (2CH_2_), 48.9 (2CH_2_). Mp: 152–154 °C. HRMS (EI^+^): *m*/*z*: calcd for C_17_H_17_N_3_F_2_ [M]^+^: 301.1391; found 301.1395.

#### 4.2.17 (*E*)-*N*-(3,4-Dichlorobenzylidene)-4-phenylpiperazin-1-amine (**1n**)

According to GP-1: 50.0 mg (282 μmol, 2.00 equiv) 4-phenylpiperazin-1-amine, 25.0 mg (141 μmol, 1.00 equiv) 3,4-dichlorobenzaldehyde, 1.0 mL toluene, stirring overnight. Addition of another 14.0 mg (80.0 μmol, 0.60 equiv) 4-phenylpiperazin-1-amine after 16 h to reach full conversion of the starting material after further stirring over a second night. Yield: 67.0 mg (90%), light yellow solid. ^1^H NMR (300 MHz, CDCl_3_): *δ* (ppm) = 7.73 (s, 1H, CH=N), 7.47 (s, 1H, Ar-H), 7.42 (s, 2H, Ar-H), 7.34–7.28 (m, 2H, Ar-H), 7.00 (d, ^3^*J* = 8.1 Hz, 2H, Ar-H), 6.95–6.90 (m, 1H, Ar-H), 3.38 (s, 8H, 4CH_2_). ^13^C NMR (75.5 MHz, CDCl_3_): *δ* (ppm) = 150.7 (Cq), 136.3 (CH=N), 133.0 (Cq-Cl), 132.8 (Cq), 131.6 (Cq-Cl), 130.4 (CHAr), 129.2 (2CHAr), 127.5 (CHAr), 125.2 (CHAr), 120.5 (CHAr), 116.7 (2CHAr), 50.8 (2CH_2_), 49.0 (2CH_2_). Mp: 176–178 °C.

#### 4.2.18 (*E*)-*N*-(2,4-Dichlorobenzylidene)-4-phenylpiperazin-1-amine (**1o**)

According to GP-1: 50.0 mg (282 μmol, 2.00 equiv) 4-phenylpiperazin-1-amine, 25.0 mg (141 μmol, 1.00 equiv) 2,4-dichlorobenzaldehyde, 1.0 mL toluene, stirring overnight. Addition of another 14.0 mg (80.0 μmol, 0.60 equiv) 4-phenylpiperazin-1-amine after 16 h to reach full conversion of the starting material after further stirring over a second night. Yield: 71.0 mg (95%), yellow solid. ^1^H NMR (300 MHz, CDCl_3_): *δ* (ppm) = 7.85 (d, ^3^*J* = 8.7 Hz, 1H, Ar-H), 7.77 (s, 1H, CH=N), 7.29 (d, ^4^*J* = 2.1 Hz, 1H, Ar-H), 7.25–7.13 (m, 3H, Ar-H), 6.94–6.91 (m, 2H, Ar-H), 6.85 (t, ^3^*J* = 7.2 Hz, 1H, Ar-H), 3.33 (s, 8H, 4 CH_2_). ^13^C NMR (75.5 MHz, CDCl_3_): *δ* (ppm) = 150.8 (Cq), 133.9 (Cq-Cl), 133.1 (Cq), 132.1 (Cq-Cl), 130.9 (CH=N), 129.3 (CHAr), 129.2 (2CHAr), 127.3 (CHAr), 127.1 (CHAr), 120.4 (CHAr), 116.7 (2CHAr), 50.9 (2CH_2_), 48.9 (2CH_2_). Mp: 113–115 °C.

#### 4.2.19 (*E*)-*N*-(2-Nitrobenzylidene)-4-phenylpiperazin-1-amine (**1p**)

According to GP-1: 40.0 mg (226 μmol, 1.00 equiv) 4-phenylpiperazin-1-amine, 26.2 mg (226 μmol, 1.00 equiv) 2-nitrobenzaldehyde, 1.0 mL toluene, stirring overnight. Addition of 3.40 mg (22.0 μmol, 0.10 equiv) 2-nitrobenzaldehyde after 20 h to reach full conversion of the starting material after further 3 h stirring. Yield: 60.0 mg (97%), yellow-brown solid. ^1^H NMR (300 MHz, CDCl_3_): *δ* (ppm) = 8.15–8.13 (m, 1H, Ar-H), 8.10 (s, 1H, CH=N), 8.00 (d, ^3^*J* = 8.1 Hz, 1H, Ar-H), 7.58 (t, ^3^*J* = 7.8 Hz, 1H, Ar-H), 7.41–7.28 (m, 3H, Ar-H), 7.01 (d, ^3^*J* = 7.8 Hz, 2H, Ar-H), 6.92 (t, ^3^*J* = 7.2 Hz, 1H, Ar-H), 3.45–3.39 (m, 8H, 4CH_2_). ^13^C NMR (75.5 MHz, CDCl_3_): *δ* (ppm) = 150.8 (Cq), 147.3 (Cq-NO_2_), 133.0 (CH=N), 131.1 (Cq), 130.2 (CHAr), 129.2 (2CHAr), 127.9 (CHAr), 127.5 (CHAr), 124.6 (CHAr), 120.4 (CHAr), 116.6 (2CHAr), 50.8 (2CH_2_), 48.8 (2CH_2_). Mp: 103–106 °C.

#### 4.2.20 (*E*)-*N*-(2-Bromobenzylidene)-4-phenylpiperazin-1-amine (**1q**)

According to GP-1: 40.0 mg (226 μmol, 1.20 equiv) 4-phenylpiperazin-1-amine, 34.8 mg (22.0 μL, 118 μmol, 1.00 equiv) 2-bromobenzaldehyde, 1.0 mL toluene, stirring overnight. Yield: 69.0 mg (89%), yellow-orange solid. ^1^H NMR (300 MHz, CDCl_3_): *δ* (ppm) = 7.95 (dd, ^3^*J* = 7.8 Hz, ^4^*J* = 1.5 Hz, 1H, Ar-H), 7.90 (s, 1H, CH=N), 7.53 (dd, ^3^*J* = 8.1 Hz, ^4^*J* = 0.9 Hz, 1H, Ar-H), 7.33–7.28 (m, 3H, Ar-H), 7.16–7.10 (m, 1H, Ar-H), 7.00 (d, ^3^*J* = 7.8 Hz, 2H, Ar-H), 6.92 (t, ^3^*J* = 7.5 Hz, 1H, Ar-H), 3.41 (s, 8H, 4 CH_2_). ^13^C NMR (75.5 MHz, CDCl_3_): *δ* (ppm) = 150.9 (Cq), 135.0 (CH=N), 134.8 (Cq), 132.8 (CHAr), 129.3 (CHAr), 129.2 (2CHAr), 127.5 (CHAr), 126.7 (CHAr), 123.2(Cq-Br), 120.3 (CHAr), 116.6 (2CHAr), 51.0 (2CH_2_), 48.9 (2CH_2_). Mp: 88–90 °C.

#### 4.2.21 (*E*)-3-((4-Phenylpiperazin-1-ylimino)methyl)benzonitrile (**1r**)

According to GP-1: 40.0 mg (226 μmol, 1.00 equiv) 4-phenylpiperazin-1-amine, 30.0 mg (226 μmol, 1.00 equiv) 3-formylbenzonitrile, 1.0 mL toluene, stirring for 6 h. Yield: 67.0 mg (99%), yellow solid. ^1^H NMR (300 MHz, CDCl_3_): *δ* (ppm) = 7.91 (s, 1H, CH=N), 7.82 (dd, ^3^*J* = 7.8 Hz, ^4^*J* = 1.2 Hz, 1H, Ar-H), 7.55–7.52 (m, 2H, Ar-H), 7.44 (t, ^3^*J* = 7.8 Hz, 1H, Ar-H), 7.33–7.28 (m, 2H, Ar-H), 7.99 (d, ^3^*J* = 7.8 Hz, 2H, Ar-H), 6.92 (t, ^3^*J* = 7.2 Hz, 1H, Ar-H), 3.39 (s, 8H, 4 CH_2_). ^13^C NMR (75.5 MHz, CDCl_3_): *δ* (ppm) = 150.8 (Cq), 137.5 (CH=N), 132.6 (CHAr), 131.0 (Cq), 130.0 (CHAr), 129.4 (CHAr), 129.3 (CHAr), 129.2 (2CHAr), 120.4 (CHAr), 118.8 (CN), 116.7 (2CHAr), 112.7 (Cq-CN), 50.8 (2CH_2_), 48.9 (2CH_2_). Mp: 140–142 °C. HRMS (EI^+^): *m*/*z*: calcd for C_18_H_18_N_4_ [M]^+^: 290.1531; found 290.1539.

#### 4.2.22 (*E*)-4-((4-Phenylpiperazin-1-ylimino)methyl)benzonitrile (**1s**)

According to GP-1: 40.0 mg (226 μmol, 1.00 equiv) 4-phenylpiperazin-1-amine, 29.6 mg (226 μmol, 1.00 equiv) 4-formylbenzonitrile, 1.0 mL toluene, stirring overnight. Yield: 60.0 mg (92%), yellow-orange solid. ^1^H NMR (300 MHz, CDCl_3_): *δ* (ppm) = 7.69 (d, ^3^*J* = 8.4 Hz, 2H, Ar-H), 7.61 (d, ^3^*J* = 8.4 Hz, 2H, Ar-H), 7.52 (s, 1H, CH=N), 7.33–7.28 (m, 2H, Ar-H), 6.99 (d, ^3^*J* = 7.8 Hz, 2H, Ar-H), 6.92 (t, ^3^*J* = 7.2 Hz, 1H, Ar-H), 3.40 (s, 8H, 4 CH_2_). ^13^C NMR (75.5 MHz, CDCl_3_): *δ* (ppm) = 150.8 (Cq), 140.6 (Cq), 132.5 (CH=N), 132.3 (2CHAr), 129.2 (2CHAr), 126.2 (2CHAr), 120.5 (CHAr), 119.1 (CN),116.7 (2CHAr), 110.8 (Cq-CN), 50.7 (2CH_2_), 48.9 (2CH_2_). Mp: 165–169 °C. HRMS (EI^+^): *m*/*z*: calcd for C_18_H_18_N_4_ [M]^+^: 290.1531; found 290.1519.

#### 4.2.23 (*E*)-*N*-(4-Methylbenzylidene)-4-phenylpiperazin-1-amine (**1t**)

According to GP-1: 40.0 mg (226 μmol, 1.00 equiv) 4-phenylpiperazin-1-amine, 27.0 mg (27.0 μL, 226 μmol, 1.00 equiv) *p*-tolylbenzaldehyde, 1.0 mL toluene, stirring overnight. Yield: 64.0 mg (99%), light yellow solid. ^1^H NMR (300 MHz, CDCl_3_): *δ* (ppm) = 7.66 (s, 1H, CH=N), 7.53 (d, ^3^*J* = 8.1 Hz, 2H, Ar-H), 7.30 (dd, ^3^*J* = 8.4 Hz, ^3^*J* = 7.5 Hz, 2H, Ar-H), 7.17 (d, ^3^*J* = 7.8 Hz, 2H, Ar-H), 7.00 (d, ^3^*J* = 8.1 Hz, 2H, Ar-H), 6.91 (t, ^3^*J* = 7.5 Hz, 1H, Ar-H), 3.41–3.34 (m, 8H, 4 CH_2_). ^13^C NMR (75.5 MHz, CDCl_3_): *δ* (ppm) = 150.9 (Cq), 138.4 (C-CH_3_), 137.3 (CH=N), 133.2 (Cq), 129.3 (2CHAr), 129.2 (2CHAr), 126.2 (2CHAr), 120.3 (CHAr), 116.6 (2CHAr), 51.3 (2CH_2_), 48.9 (2CH_2_), 21.3 (CH_3_). Mp: 184–186 °C.

#### 4.2.24 (*E*)-*N*-(4-(Dimethylamino)benzylidene)-4-phenylpiperazin-1-amine (**1u**)

According to GP-1: 40.0 mg (226 μmol, 1.00 equiv) 4-phenylpiperazin-1-amine, 34.0 mg (226 μmol, 1.00 equiv) 4-dimethylaminobenzaldehyde, 1.0 mL toluene, stirring overnight. Addition of another 4.00 mg (23.0 μmol, 0.10 equiv) 4-phenylpiperazin-1-amine after 18 h to reach full conversion of the starting material after further 2 h stirring. Yield: 43.0 mg (61%), light yellow solid. ^1^H NMR (300 MHz, CDCl_3_): *δ* (ppm) = 7.68 (br s, 1H, CH=N), 7.53 (d, ^3^*J* = 8.7 Hz, 2H, Ar-H), 7.33–7.28 (m, 2H, Ar-H), 7.00 (d, ^3^*J* = 8.1 Hz, 2H, Ar-H), 6.90 (t, ^3^*J* = 7.2 Hz, 1H, Ar-H), 6.71 (d, ^3^*J* = 8.7 Hz, 2H, Ar-H), 3.40–3.38 (m, 4H, 2CH_2_), 3.31–3.29 (m, 4H, 2CH_2_), 2.99 (s, 6H, 2CH_3_). ^13^C NMR (75.5 MHz, CDCl_3_): *δ* (ppm) = 151.0 (Cq-N), 150.8 (Cq), 139.5 (CH=N), 129.1 (2CHAr), 127.6 (2CHAr), 124.2 (Cq), 120.0 (CHAr), 116.4 (2CHAr), 112.1 (2CHAr), 51.8 (2CH_2_), 48.9 (2CH_2_), 40.4 (2CH_3_). Mp: 190 °C (dec.).

#### 4.2.25 (*E*)-*N*-(4-((4-Phenylpiperazin-1-ylimino)methyl)phenyl)acetamide (**1v**)

According to GP-1: 30.0 mg (169 μmol, 1.00 equiv) 4-phenylpiperazin-1-amine, 27.5 mg (169 μmol, 1.00 equiv) 4-acetamidobenzaldehyde, 1.0 mL toluene, stirring overnight. Yield: 54.0 mg (96%), yellow solid. ^1^H NMR (300 MHz, DMSO-*d*_6_): *δ* (ppm) = 10.00 (s, 1H, NH), 7.68 (s, 1H, CH=N), 7.61–7.50 (m, 4H, Ar-H), 7.26–7.21 (m, 2H, Ar-H), 7.00 (d, ^3^*J* = 8.1 Hz, 2H, Ar-H), 6.81 (t, ^3^*J* = 7.2 Hz, 1H, Ar-H), 3.32–3.30 (m, 4H, 2CH_2_), 3.23–3.21 (m, 4H, 2CH_2_), 2.05 (s, 3H, CH_3_). ^13^C NMR (75.5 MHz, DMSO-*d*_6_): *δ* (ppm) = 168.1 (C=O), 150.6 (Cq), 139.1 (Cq-NH), 136.0 (CH=N), 130.9 (Cq), 128.8 (2CHAr), 126.2 (2CHAr), 119.1 (CHAr), 118.8 (2CHAr), 115.8 (2CHAr), 50.7 (2CH_2_), 47.7 (2CH_2_), 23.9 (CH_3_). Mp: 229–232 °C. HRMS (EI^+^): *m*/*z*: calcd for C_19_H_22_ON_4_ [M]^+^: 322.1794; found 322.1795.

#### 4.2.26 (*E*)-2-(Methyl(4-((4-phenylpiperazin-1-ylimino)methyl)phenyl)amino)ethanol (**1w**)

According to GP-1: 30.0 mg (169 μmol, 1.00 equiv) 4-phenylpiperazin-1-amine, 30.3 mg (169 μmol, 1.00 equiv) *N*-methyl-*N*-(2-hydroxyethyl)-4-aminobenzaldehyde, 1.0 mL toluene, stirring overnight. Yield: 48.0 mg (84%), light yellow solid. ^1^H NMR (300 MHz, DMSO-*d*_6_): *δ* (ppm) = 7.66 (s, 1H, CH=N), 7.42 (d, ^3^*J* = 8.7 Hz, 2H, Ar-H), 7.23 (t, ^3^*J* = 8.1 Hz, 2H, Ar-H), 6.99 (d, ^3^*J* = 8.1 Hz, 2H, Ar-H), 6.81 (t, ^3^*J* = 7.2 Hz, 1H, Ar-H), 6.68 (d, ^3^*J* = 9.0 Hz, 2H, Ar-H), 4.68 (t, ^3^*J* = 5.1 Hz, OH), 3.58–3.52 (m, 2H, CH_2_), 3.43–3.39 (m, 2H, CH_2_), 3.32–3.38 (m, 4H, 2CH_2_), 3.17–3.14 (m, 4H, 2CH_2_), 2.95 (s, 3H, N-CH_3_). ^13^C NMR (75.5 MHz, DMSO-*d*_6_): *δ* (ppm) = 150.6 (Cq-N), 149.2 (Cq), 138.0 (CH=N), 128.8 (2CHAr), 127.1 (2CHAr), 123.4 (Cq), 119.0 (CHAr), 115.7 (2CHAr), 111.3 (2CHAr), 58.0 (N-CH_2_), 54.0 (O-CH_2_), 51.2 (2CH_2_), 47.8 (2CH_2_), 38.5 (NCH_3_). Mp: 202–204 °C. HRMS (EI^+^): *m*/*z*: calcd for C_20_H_26_ON_4_ [M]^+^: 338.2107; found 338.2117.

#### 4.2.27 (*E*)-*N*-(Benzo[*d*][1,3]dioxol-5-ylmethylen)-4-phenylpiperazin-1-amine (**1x**)

According to GP-1: 38.0 mg (215 μmol, 1.00 equiv) 4-phenylpiperazin-1-amine, 1.0 mL toluene, 32.2 mg (215 μmol, 1.00 equiv) benzo[*d*][1,3]-dioxol-5-carbaldehyde. Stirring at 100 °C for 4 h. Final purification by recrystallization from 1.0 mL toluene produced the pure product. Yield: 40.0 mg (67%), orange-brown solid. ^1^H NMR (300 MHz, DMSO-*d*_6_): *δ* (ppm) = 7.67 (s, 1H, CH=N), 7.26–7.21 (m, 2H, Ar-H), 7.18–7.17 (m, 1H, Ar-H), 7.05–6.99 (m, 3H, Ar-H), 6.92–6.90 (m, 1H, Ar-H), 6.83–6.79 (m, 1H, Ar-H), 6.03 (s, 2H, CH_2_), 3.31 (m, 4H, 2CH_2_), 3.21 (m, 4H, 2CH_2_). ^13^C NMR (75.5 MHz, DMSO-*d*_6_): *δ* (ppm) = 150.6 (Cq), 147.6 (Cq), 147.2 (Cq), 136.1 (CH=N), 130.7 (Cq), 128.8 (2CHAr), 121.0 (CHAr), 119.1 (CHAr), 115.8 (2CHAr), 108.1 (CHAr), 104.3 (CHAr), 101.0 (CH_2_), 50.8 (2CH_2_), 47.7 (2CH_2_). Mp: 125–129 °C.

#### 4.2.28 (*E*)-*N*-((2,3-Dihydrobenzol[*b*][1,4]dioxin-6-yl)methylen)-4-phenylpiperazin-1-amine (**1y**)

According to GP-1: 50.0 mg (282 μmol, 1.00 equiv) 4-phenylpiperazin-1-amine, 46.0 mg (282 μmol, 1.00 equiv) 1,4-benzodioxan-6-carboxaldehyde, 1.0 mL toluene, stirring overnight. Yield: 87.0 mg (95%), light brown solid. ^1^H NMR (300 MHz, DMSO-*d*_6_): *δ* (ppm) = 7.63 (s, 1H, CH=N), 7.26–7.21 (m, 2H, Ar-H), 7.09–7.06 (m, 2H, Ar-H), 7.01–6.98 (m, 2H, Ar-H), 6.86–6.79 (m, 2H, Ar-H), 4.24 (s, 4H, 2CH_2_), 3.32–3.29 (m, 4H, 2CH_2_), 3.21–3.17 (s, 4H, 2CH_2_). ^13^C NMR (75.5 MHz, DMSO-*d*_6_): *δ* (ppm) = 150.6 (Cq-O), 143.5 (Cq), 143.3 (Cq-O), 135.9 (CH=N), 129.7 (Cq), 128.8 (2CHAr), 119.1 (CHAr), 117.0 (CHAr), 115.8 (2CHAr), 114.0 (CHAr), 64.0 (2CH_2_), 50.8 (2CH_2_), 47.7 (2CH_2_). Mp: 186–187 °C. HRMS (EI^+^): *m*/*z*: calcd for C_19_H_21_O_2_N_3_ [M]^+^: 323.1634; found 323.1644.

#### 4.2.29 (*E*)-5-((4-Phenylpiperazin-1-ylimino)methyl)-1*H*-benzo[*d*]imidazol-2(3*H*)-one (**1z**)

According to GP-1: 65.6 mg (370 μmol, 1.00 equiv) 4-phenylpiperazin-1-amine, 1.0 mL toluene, 60.0 mg (370 μmol, 1.00 equiv) 2-oxo-2,3-dihydro-1*H*-benzimidazol-5-carbaldehyde. Stirring at 100 °C for 5 h. Additional 6.10 mg (34.0 μmol, 0.09 equiv) 4-phenylpiperazin-1-amine and further stirring for 3 h were necessary to reach full conversion of aldehyde. Yield: 119.0 mg (99%), colorless solid. *R_f_* (EtOAc/MeOH 30:1): 0.27. ^1^H NMR (300 MHz, DMSO-*d*_6_): *δ* (ppm) = 10.64 (br s, 2H, 2NH), 7.73 (s, 1H, CH=N), 7.26–7.21 (m, 3H, Ar-H), 7.18–7.14 (m, 1H, Ar-H), 7.01 (d, ^3^*J* = 8.1 Hz, 2H, Ar-CH), 6.90 (d, ^3^*J* = 8.1 Hz, 1H, Ar-H), 6.81 (t, ^3^*J* = 7.2 Hz, 1H, Ar-H), 3.33–3.30 (m, 4H, 2CH_2_), 3.22–3.20 (m, 4H, 2CH_2_). ^13^C NMR (75.5 MHz, DMSO-*d*_6_): *δ* (ppm) = 155.4 (C=O), 150.6 (Cq), 137.4 (CH=N), 130.0 (Cq), 129.1 (Cq), 128.9 (2CHAr), 119.9 (CHAr), 119.1 (CHAr), 115.8 (2CHAr), 115.3 (Cq), 108.2 (CHAr), 104.9 (CHAr), 50.9 (2CH_2_), 47.8 (2CH_2_). Mp: 320–330 °C (dec.). HRMS (EI^+^): *m*/*z*: calcd for C_18_H_19_ON_5_ [M]^+^: 321.1590; found 321.1606.

#### 4.2.30 (*E*)-*N*-Pentyliden-4-phenylpiperazin-1-amine (**2a**)

According to GP-1: 50.0 mg (282 μmol, 1.00 equiv) 4-phenylpiperazin-1-amine, 24.3 mg (30.0 μL, 282 μmol, 1.00 equiv) valeraldehyde, 1.0 mL toluene, stirring overnight. Yield: 65.0 mg (94%), orange oil. ^1^H NMR (300 MHz, CDCl_3_): *δ* (ppm) = 7.31–7.25 (m, 2H, Ar-H), 7.05 (t, ^3^*J* = 5.4 Hz, 1H, CH=N), 6.96 (d, ^3^*J* = 7.8 Hz, 2H, Ar-H), 6.88 (t, ^3^*J* = 7.2 Hz, 1H, Ar-H), 3.34 (t, ^3^*J* = 5.1 Hz, 4H, 2CH_2_), 3.12 (t, ^3^*J* = 5.1 Hz, 4H, 2CH_2_), 2.28 (q, ^3^*J* = 7.2 Hz, 2H, CH_2_), 1.55–1.32 (m, 4H, 2CH_2_), 0.93 (t, ^3^*J* = 7.2 Hz, 3H, CH_3_). ^13^C NMR (75.5 MHz, CDCl_3_): *δ* (ppm) = 150.9 (CH=N), 143.5 (Cq), 129.1 (2CHAr), 120.1 (CHAr), 116.4 (2CHAr), 51.9 (2CH_2_), 48.8 (2CH_2_), 32.8 (CH_2_), 29.5 (CH_2_), 22.3 (CH_2_), 13.9 (CH_3_). HRMS (EI^+^): *m*/*z*: calcd for C_15_H_23_N_3_ [M]^+^: 245.1892; found 245.1885.

#### 4.2.31 (*E*)-*N*-((*E*)-Hex-2-enyliden)-4-phenylpiperazin-1-amine (**2b**)

According to GP-1: 51.1 mg (289 μmol, 1.00 equiv) 4-phenylpiperazin-1-amine, 28.3 mg (30.0 μL, 289 μmol, 1.00 equiv) trans-2-hexen-1-al, 3.0 mL toluene, stirring for 3 h. Yield: 74.3 mg (>99%), brown solid. ^1^H NMR (300 MHz, CDCl_3_): *δ* (ppm) = 7.38 (d, ^3^*J* = 8.7 Hz, 1H, CH=N), 7.31–7.27 (m, 2H, Ar-H), 6.98 (d, ^3^*J* = 7.8 Hz, 2H, Ar-H), 6.89 (t, ^3^*J* = 7.2 Hz, 1H, Ar-H), 6.28–6.20 (m, 1H, CH), 5.99–5.89 (m, 1H, CH), 3.36–3.33 (m, 4H, 2CH_2_), 3.22–3.19 (m, 4H, 2CH_2_), 2.20–2.12 (m, 2H, CH_2_), 1.47 (m, 2H, CH_2_), 0.93 (t, ^3^*J* = 7.2 Hz, 3H, CH_3_). ^13^C NMR (75.5 MHz, CDCl_3_): *δ* (ppm) = 150.9 (Cq), 140.6 (CH), 138.4 (CH=N), 129.1 (2CHAr), 128.7 (CH), 120.1 (CHAr), 116.5 (2CHAr), 51.3 (2CH_2_), 48.8 (2CH_2_), 34.7 (CH_2_), 22.1 (CH_2_), 13.6 (CH_3_). Mp: 48 °C. HRMS (EI^+^): *m*/*z*: calcd for C_16_H_23_N_3_ [M]^+^: 257.1892; found 257.1898.

#### 4.2.32 (*E*)-*N*-(Cyclohexylmethylen)-4-phenylpiperazin-1-amine (**2c**)

According to GP-1: 40.0 mg (226 μmol, 1.00 equiv) 4-phenylpiperazin-1-amine, 25.1 mg (27.1 μL, 226 μmol, 1.00 equiv) cyclohexanecarboxaldehyde, 1.0 mL toluene, stirring for 4 h. Yield: 60.0 mg (98%), yellow-brown solid. ^1^H NMR (300 MHz, CDCl_3_): d (ppm) = 7.31–7.24 (m, 3H, Ar-H, CH=N), 6.96 (d, ^3^*J* = 8.1 Hz, 2H, Ar-H), 6.90–6.86 (m, 1H, Ar-H), 3.35–3.32 (m, 4H, 2CH_2_), 3.12–3.08 (m, 4H, 2CH_2_), 2.25–2.21 (m, 1H, CH), 1.82–1.66 (m, 5H, CH_2_, CH), 1.35–1.16 (m, 5H, CH_2_, CH). ^13^C NMR (75.5 MHz, CDCl_3_): *δ* (ppm) = 150.9 (CH=N), 147.4 (Cq), 129.1 (2CHAr), 120.0 (CHAr), 116.4 (2CHAr), 51.8 (2CH_2_), 48.8 (2CH_2_), 41.4 (CH), 31.0 (2CH_2_), 26.0 (CH_2_), 25.6 (2CH_2_). Mp: 69–71 °C. HRMS (EI^+^): *m*/*z*: calcd for C_17_H_25_N_3_ [M]^+^: 271.2048; found 271.2058.

#### 4.2.33 (*E*)-*N*-(Cyclohexenylmethylen)-4-phenylpiperazin-1-amine (**2d**)

According to GP-1: 40.0 mg (226 μmol, 1.00 equiv) 4-phenylpiperazin-1-amine, 24.9 mg (25.8 μL, 226 μmol, 1.00 equiv) 1-cyclohexene-1-carboxaldehyde, 1.0 mL toluene, stirring for 4 h. Yield: 57.0 mg (94%), yellow-brown solid. ^1^H NMR (300 MHz, CDCl_3_): *δ* (ppm) = 7.34 (s, 1H, CH=N), 7.31–7.26 (m, 2H, Ar-H), 6.97 (d, ^3^*J* = 8.1 Hz, 2H, Ar-H), 6.88 (t, ^3^*J* = 7.2 Hz, 1H, Ar-H), 5.92 (t, ^3^*J* = 3.9 Hz, 1H, CH), 3.36–3.33 (m, 4H, 2CH_2_), 3.20–3.17 (m, 4H, 2CH_2_), 2.31 (br s, 2H, CH_2_), 2.19 (br s, 2H, CH_2_), 1.67–1.65 (m, 4H, 2CH_2_). ^13^C NMR (75.5 MHz, CDCl_3_): *δ* (ppm) = 151.9 (Cq), 142.5 (CH=N), 136.3 (Cq), 132.3 (CH), 129.1 (2CHAr), 120.0 (CHAr), 116.4 (2CHAr), 51.5 (2CH_2_), 48.9 (2CH_2_), 25.9 (CH_2_), 23.8 (CH_2_), 22.7 (CH_2_), 22.2 (CH_2_). Mp: 118–120 °C. HRMS (EI^+^): *m*/*z*: calcd for C_17_H_23_N_3_ [M]^+^: 269.1892; found 269.1899.

#### 4.2.34 (*E*)-4-Phenyl-*N*-(pyridine-2-ylmethylen)piperazin-1-amine (**2e**)

According to GP-1: 50.0 mg (282 μmol, 2.00 equiv) 4-phenylpiperazin-1-amine, 30.2 mg (27.0 μL, 282 μmol, 1.00 equiv) 2-pyridinecarboxaldehyde, 1.0 mL toluene, stirring for 4.5 h. Additional 3.0 mg (28.0 μmol, 0.10 equiv) 2-pyridinecarboxaldehyde and further stirring overnight were necessary to reach full conversion of amine. Yield: 65.0 mg (87%), brown solid. ^1^H NMR (300 MHz, CDCl_3_): *δ* (ppm) = 8.38–8.36 (m, 1H, Ar-H), 7.71–7.69 (m, 1H, Ar-H), 7.51 (s, 1H, CH=N), 7.15–7.08 (m, 3H, Ar-H), 7.00–6.97 (m, 1H, Ar-H), 6.81 (d, ^3^*J* = 7.8 Hz, 2H, Ar-H), 6.73 (t, ^3^*J* = 7.2 Hz, 1H, Ar-H), 3.23–3.20 (m, 8H, 4CH_2_). ^13^C NMR (75.5 MHz, CDCl_3_): *δ* (ppm) = 155.2 (Cq), 150.9 (Cq), 149.0 (CHAr), 136.3 (CH=N), 135.9 (CHAr), 129.2 (2CHAr), 122.4 (CHAr), 120.3 (CHAr), 119.3 (CHAr), 116.6 (2CHAr), 50.7 (2CH_2_), 48.9 (2CH_2_). Mp: 117-119 °C.

#### 4.2.35 (*E*)-4-Phenyl-*N*-(pyridine-3-ylmethylen)piperazin-1-amine (**2f**)

According to GP-1: 50.0 mg (282 μmol, 1.00 equiv) 4-phenylpiperazin-1-amine, 30.2 mg (26.5 μL, 282 μmol, 1.00 equiv) 3-pyridinecarboxaldehyde, 1.0 mL toluene, stirring for 5 h. Yield: 75.0 mg (99%), red-brown solid. ^1^H NMR (300 MHz, CDCl_3_): *δ* (ppm) = 8.75 (d, ^4^*J* = 1.8 Hz, 1H, Ar-H), 8.51–8.49 (m, 1H, Ar-H), 8.02–7.98 (m, 1H, Ar-H), 7.56 (s, 1H, CH=N), 7.33–7.25 (m, 3H, Ar-H), 6.99 (d, ^3^*J* = 8.1 Hz, 2H, Ar-H), 6.91 (t, ^3^*J* = 7.2 Hz, 1H, Ar-H), 3.39 (s, 8H, 4 CH_2_). ^13^C NMR (75.5 MHz, CDCl_3_): d (ppm) = 150.8 (Cq), 149.0 (CHAr), 148.2 (CHAr), 132.3 (CH=N), 132.2 (CHAr), 132.0 (Cq), 129.2 (2CHAr), 123.5 (CHAr), 120.4 (CHAr), 116.6 (2CHAr), 50.8 (2CH_2_), 48.9 (2CH_2_). Mp: 111–113 °C

#### 4.2.36 (*E*)-4-Phenyl-*N*-(pyridine-4-ylmethylen)piperazin-1-amine (**2g**)

According to GP-1: 50.0 mg (282 μmol, 1.00 equiv) 4-phenylpiperazin-1-amine, 30.2 mg (26.5 μL, 282 μmol, 1.00 equiv) 4-pyridinecarboxaldehyde, 1.0 mL toluene, stirring for 5 h. Yield: 75.0 mg (99%), orange solid. ^1^H NMR (300 MHz, CDCl_3_): *δ* (ppm) = 8.55 (d, ^3^*J* = 6.0 Hz, 2H, Ar-H), 7.46 (d, ^3^*J* = 6.0 Hz, 2H, Ar-H), 7.43 (s, 1H, CH=N), 7.33–7.27 (m, 2H, Ar-H), 6.98 (d, ^3^*J* = 7.8 Hz, 2H, Ar-H), 6.92 (t, ^3^*J* =7.2 Hz, 1H, Ar-H), 3.41–3.40 (m, 8H, 4 CH2). ^13^C NMR (75.5 MHz, CDCl_3_): *δ* (ppm) = 150.8 (Cq), 149.8 (2CHAr), 143.6 (Cq), 131.6 (CH=N), 129.2 (2CHAr), 120.5 (CHAr), 120.1 (2CHAr), 116.7 (2CHAr), 50.6 (2CH_2_), 48.9 (2CH_2_). Mp: 156–158 °C.

#### 4.2.37 (*E*)-*N*-(Naphthalene-2-ylmethylen)-4-phenylpiperazin-1-amine (**2h**)

According to GP-1: 40.0 mg (226 μmol, 2.00 equiv) 4-phenylpiperazin-1-amine, 35.3 mg (226 μmol, 1.00 equiv) 2-naphthaldehyde, 1.0 mL toluene, stirring overnight. Addition of 2.0 mg (13.0 μmol, 0.06 equiv) 4-phenylpiperazin-1-amine after 18 h to reach full conversion of the starting material after further stirring for 6 h. Yield: 61.0 mg (82%), yellow solid. ^1^H NMR (300 MHz, CDCl_3_): *δ* (ppm) = 7.98–7.96 (m, 1H, Ar-H), 7.88 (s, 1H, CH=N), 7.84–7.80 (m, 4H, Ar-H), 7.48–7.46 (m, 2H, Ar-H), 7.34–7.29 (m, 2H, Ar-H), 7.02 (d, ^3^*J* = 8.1 Hz, 2H, Ar-H), 6.95–6.90 (m, 1H, Ar-H), 3.42 (s, 8H, 4CH_2_). ^13^C NMR (75.5 MHz, CDCl_3_): *δ* (ppm) = 150.9 (Cq), 136.6 (CH=N), 133.8 (Cq), 133.5 (Cq), 133.4 (Cq), 129.2 (2CHAr), 128.3 (CHAr), 128.0 (CHAr), 127.8 (CHAr), 126.6 (CHAr), 126.2 (CHAr), 126.0 (CHAr), 123.1 (CHAr), 120.3 (CHAr), 116.6 (2CHAr), 51.2 (2CH_2_), 49.0 (2CH_2_). Mp: 195–198 °C (dec.). HRMS (EI^+^): *m*/*z*: calcd for C_21_H_21_N_3_ [M]^+^: 315.1736; found 315.1738.

#### 4.2.38 (*E*)-4-Phenyl-*N*-(quinoline-2-ylmethylen)-piperazin-1-amine (**2i**)

According to GP-1: 40.0 mg (226 μmol, 1.00 equiv) 4-phenylpiperazin-1-amine, 35.5 mg (226 μmol, 1.00 equiv) 2-quinolinecarboxaldehyde, 1.0 mL toluene, stirring for 5.5 h. Yield: 59.0 mg (83%), dark yellow solid. ^1^H NMR (300 MHz, CDCl_3_): d (ppm) = 8.09 (s, 2H, Ar-H), 8.05 (d, ^3^*J* = 8.4 Hz, 1H, Ar-H), 7.86 (s, 1H, CH=N), 7.78 (dd, ^3^*J* = 8.1 Hz, ^4^*J* = 0.6 Hz, 1H, Ar-H), 7.72–7.67 (m, 1H, Ar-H), 7.52–7.47 (m, 1H, Ar-H), 7.33–7.28 (m, 2H, Ar-H), 7.00 (d, ^3^*J* = 7.8 Hz, 2H, Ar-H), 6.92 (t, ^3^*J* = 7.2 Hz, 1H, Ar-H), 3.52–3.49 (m, 4H, 2CH_2_), 3.43–3.39 (m, 4H, 2CH_2_). ^13^C NMR (75.5 MHz, CDCl_3_): *δ* (ppm) = 155.6 (Cq), 150.8 (Cq), 147.7 (Cq), 136.1 (CH=N), 135.7 (Cq), 129.6 (CHAr), 129.2 (2CHAr), 128.7 (CHAr), 127.7 (CHAr), 127.6 (CHAr), 126.2 (CHAr), 120.4 (CHAr), 117.5 (CHAr), 116.7 (2CHAr), 50.7 (2CH_2_), 48.9 (2CH_2_). Mp: 210–212 °C. HRMS (EI^+^): *m*/*z*: calcd for C_20_H_20_N_4_ [M]^+^: 316.1688; found 316.1696.

#### 4.2.39 (*E*)-*N*-(Naphthalene-1-methylen)-4-phenylpiperazin-1-amine (**2j**)

According to GP-1: 50.9 mg (288 μmol, 1.00 equiv) 4-phenylpiperazin-1-amine, 44.9 mg (39.0 μL, 288 μmol, 1.00 equiv) 1-naphthaldehyde, 3.0 mL toluene, stirring for 4 h. Yield: 88.6 mg (98%), brown solid. ^1^H NMR (300 MHz, CDCl_3_): *δ* (ppm) = 8.60 (d, ^3^*J* = 8.1 Hz, 1H, Ar-H), 8.33 (s, 1H, CH=N), 7.91–7.87 (m, 2H, Ar-H), 7.83 (d, 3 J = 8.1 Hz, 1H, Ar-H), 7.59–7.48 (m, 3H, Ar-H), 7.36–7.30 (m, 2H, Ar-H), 7.03 (d, ^3^*J* = 7.8 Hz, 2H, Ar-H), 6.94 (t, ^3^*J* = 7.2 Hz, 1H, Ar-H), 3.47 (s, 8H, 4CH_2_). ^13^C NMR (75.5 MHz, CDCl_3_): *δ* (ppm) = 150.9 (Cq), 135.7 (CH=N), 133.9 (Cq), 131.5 (Cq), 130.7 (Cq), 129.2 (2CHAr), 128.8 (CHAr), 128.7 (CHAr), 126.3 (CHAr), 125.7 (CHAr), 125.5 (CHAr), 125.3 (CHAr), 123.8 (CHAr), 120.3 (CHAr), 116.6 (2CHAr), 51.3 (2CH_2_), 49.0 (2CH_2_). Mp: 118 °C.

#### 4.2.40 (*E*)-4-((4-(Pyridine-4-yl)piperazin-1-ylimino)methyl)benzene-1,3-diol (**3a**)

According to GP-1: 250 mg (1.40 mmol, 1.00 equiv) 4-(pyridine-4-yl)piperazine-1-amine), 4.0 mL toluene, 190 mg (1.40 mmol, 1.00 equiv) 2,4-dihydroxybenzaldehyde. Stirring at 100 °C for 2 h. Trituration with 15 mL hot toluene yielded the pure product. Yield: 310.0 mg (74%), brown solid. *R_f_* (MeOH): 0.30. ^1^H NMR (300 MHz, DMSO-*d*_6_): *δ* (ppm) = 11.52 (br s, 1H, OH), 9.90 (br s, 1H, OH), 8.19 (br s, 2H, Ar-H), 7.97 (s, 1H, CH=N), 7.17 (d, ^3^*J* = 8.4 Hz, 1H, Ar-H), 6.88 (d, ^3^*J* = 6.0 Hz, 2H, Ar-H), 6.31 (dd, ^3^*J* = 8.4 Hz, ^4^*J* = 2.1 Hz, 1H, Ar-H), 6.24 (d, ^4^*J* = 2.1 Hz, 1H, Ar-H), 3.53–3.49 (m, 4H, 2CH_2_), 3.15–3.12 (m, 4H, 2CH_2_). ^13^C NMR (75.5 MHz, DMSO-*d*_6_): *δ* (ppm) = 159.1 (Cq-OH), 158.6 (Cq-OH), 154.1 (Cq), 149.8 (2CHAr), 142.7 (CH=N), 130.8 (CHAr), 111.4 (Cq), 108.6 (2CHAr), 107.0 (CHAr), 102.4 (CHAr), 50.7 (2CH_2_), 44.6 (2CH_2_). Mp: 270–273 °C (dec.). HRMS (EI^+^): *m*/*z*: calcd for C_16_H_18_O_2_N_4_ [M]^+^: 298.1430; found 298.1447.

#### 4.2.41 (*E*)-4-((4-(4-Fluorophenyl)piperazin-1-ylimino)methyl)benzene-1,3-diol (**3b**)

According to GP-1: 150 mg (768 μmol, 1.00 equiv) 4-(4-fluorophenyl)piperazine-1-amine, 2.0 mL toluene, 110 mg (768 μmol, 1.00 equiv) 2,4-dihydroxybenzaldehyde. Stirring at 100 °C for 2 h. Recrystallization from 7 mL toluene yielded the pure product. Yield: 67.8 mg (25%), brown solid. *R_f_* (MeOH): 0.64. ^1^H NMR (300 MHz, DMSO-*d*_6_): *δ* (ppm) = 11.59 (br s, 1H, OH), 9.72 (br s, 1H, OH), 7.95 (s, 1H, CH=N), 7.17 (d, ^3^*J* = 8.4 Hz, 1H, Ar-H), 7.10–6.99 (m, 4H, Ar-H), 6.31 (dd, ^3^*J* = 8.4 Hz, ^4^*J* = 2.4 Hz, 1H, Ar-H), 6.25 (d, ^4^*J* = 2.4 Hz, 1H, Ar-H), 3.26–3.25 (m, 4H, 2CH_2_), 3.18–3.16 (m, 4H, 2CH_2_). ^13^C NMR (75.5 MHz, DMSO-*d*_6_): *δ* (ppm) = 159.0 (Cq-OH), 158.6 (Cq-OH), 157.7, 154.6 (^1^*J*_C–F_ = 236.0 Hz, Cq-F), 147.4, 147.3 (^4^*J*_C–F_ = 2.0 Hz, Cq), 142.3 (CH=N), 130.7 (CHAr), 117.7, 117.6 (^3^*J*_C–F_ = 7.6 Hz, 2CHAr), 115.4, 115.1 (^2^*J*_C–F_ = 21.9 Hz, 2CHAr), 111.4 (Cq), 107.0 (CHAr), 102.4 (CHAr), 51.1 (2CH_2_), 48.3 (2CH_2_). Mp: 176–178 °C.

#### 4.2.42 (*E*)-4-((4-*m*-Tolylpiperazin-1-ylimino)methyl)benzene-1,3-diol (**3c**)

According to GP-1: 150 mg (731 μmol, 1.00 equiv) 4-*m*-tolylpiperazine-1-amine, 2.0 mL toluene, 101 mg (731 μmol, 1.00 equiv) 2,4-dihydroxybenzaldehyde. Stirring at 100 °C for 2 h. Recrystallization from 2 mL toluene yielded the pure product. Yield: 141.8 mg (58%), brown solid. *R_f_* (MeOH): 0.65. ^1^H NMR (300 MHz, DMSO- *d*_6_): *δ* (ppm) = 11.60 (s, 1H, OH), 9.75 (br s, 1H, OH), 7.95 (s, 1H, CH=N), 7.18–7.09 (m, 2H, Ar-H), 6.82–6.78 (m, 2H, Ar-H), 6.64 (d, ^3^*J* = 7.2 Hz, 1H, Ar-H), 6.31 (dd, ^3^*J* = 8.1, ^4^*J* = 2.1 Hz, 1H, Ar-H), 6.25 (d, ^4^*J* = 2.1 Hz, 1H, Ar-H), 3.31–3.28 (m, 4H, 2CH_2_), 3.17–3.15 (m, 4H, 2CH_2_), 2.26 (s, 3H, CH_3_). ^13^C NMR (75.5 MHz, DMSO-*d*_6_): *δ* (ppm) = 159.0 (Cq-OH), 158.6 (Cq-OH), 150.5 (Cq), 142.3 (CH=N), 138.0 (Cq-CH_3_), 130.7 (CHAr), 128.7 (CHAr), 120.0 (CHAr), 116.4 (CHAr), 113.0 (CHAr), 111.4 (Cq), 107.0 (CHAr), 102.4 (CHAr), 51.1 (2CH_2_), 47.6 (2CH_2_), 21.3 (CH_3_). Mp: 135–139 °C. HRMS (EI^+^): *m*/*z*: calcd for C_18_H_21_O_2_N_3_ [M]^+^: 311.1654; found 311.1634.

#### 4.2.43 (*E*)-4-((4-Benzylpiperazin-1-ylimino)methyl)benzene-1,3-diol (**3d**)

According to GP-1: 150 mg (785 μmol, 1.00 equiv) 4-benzylpiperazin-1-amine, 2.0 mL toluene, 108 mg (785 μmol, 1.00 equiv) 2,4-dihydroxybenzaldehyde. Stirring at 100 °C for 2 h. Final purification by recrystallization from 4.0 mL toluene yielded the pure product. Yield: 134.0 mg (95%), beige solid. ^1^H NMR (300 MHz, DMSO-*d*_6_): *δ* (ppm) = 11.61 (br s, 1H, OH), 9.67 (br s, 1H, OH), 7.83 (s, 1H, CH), 7.33–7.25 (m, 5H, Ar-H), 7.12 (d, ^3^*J* = 8.4 Hz, 1H, Ar-H), 6.30 (dd, ^3^*J* = 8.4 Hz, ^4^*J* = 2.1 Hz, 1H, Ar-H), 6.23 (d, ^4^*J* = 2.1 Hz, 1H, Ar-H), 3.52 (s, 2H, CH_2_), 3.03 (s, 4H, 2CH_2_), 2.53 (s, 4H, 2CH_2_). ^13^C NMR (75.5 MHz, DMSO-*d*_6_): *δ* (ppm) = 158.8 (Cq-OH), 158.5 (Cq-OH), 141.3 (CH=N), 137.9 (Cq), 130.6 (CHAr), 128.7 (2CHAr), 128.1 (2CHAr), 126.9 (CHAr), 111.5 (Cq), 106.9 (CHAr), 102.3 (CHAr), 61.6 (CH_2_), 51.4 (2CH_2_), 51.1 (2CH_2_). Mp: 225–228 °C. HRMS (EI^+^): *m*/*z*: calcd for C_18_H_21_O_2_N_3_ [M]^+^: 311.1634; found 311.1656.

#### 4.2.44 (*E*)-4-((4-Phenethylpiperazin-1-ylimino)methyl)benzene-1,3-diol (**3e**)

According to GP-1: 100 mg (488 μmol, 1.00 equiv, 90% purity) 4-phenethylpiperazin-1-amine, 2.0 mL toluene, 60.7 mg (439 μmol, 1.00 equiv) 2,4-dihydroxybenzaldehyde. Stirring at 100 °C overnight. Final purification by recrystallization from 20.0 mL toluene yielded the pure product. Yield: 148.5 mg (94%), orange-brown solid. ^1^H NMR (300 MHz, DMSO- *d*_6_): *δ* (ppm) = 11.83 (br s, 1H, OH), 9.88 (br s, 1H, OH), 8.05 (s, 1H, CH=N), 7.51–7.32 (m, 6H, Ar-H), 6.50 (dd, ^3^*J* = 8.4 Hz, ^4^*J* = 1.8 Hz, 1H, Ar-H), 6.43 (d, ^4^*J* = 1.8 Hz, 1H, Ar-H), 3.23 (br s, 2H, CH_2_), 2.98–2.93 (m, 2H, CH_2_), 2.83–2.70 (m, 8H, 4CH_2_). ^13^C NMR (75.5 MHz, DMSO-*d*_6_): *δ* (ppm) = 158.8 (Cq-OH), 158.5 (Cq-OH), 141.3 (CH=N), 140.2 (Cq), 130.6 (CHAr), 128.6 (2CHAr), 128.1 (2CHAr), 125.8 (CHAr), 111.5 (Cq), 106.9 (CHAr), 102.3 (CHAr), 59.2 (CH_2_), 51.6 (2CH_2_), 51.1 (2CH_2_), 32.8 (CH_2_). Mp: 211–214 °C. HRMS (EI^+^): *m*/*z*: calcd for C_19_H_23_O_2_N_3_ [M]^+^: 325.1790; found 325.1799.

#### 4.2.45 (*E*)-4-((4-Cyclohexylpiperazin-1-ylimino)methyl)benzene-1,3-diol (**3f**)

According to GP-1: 150 mg (818 μmol, 1.00 equiv) 4-cyclohexylpiperazine-1-amine, 2.0 mL toluene, 113 mg (818 μmol, 1.00 equiv) 2,4-dihydroxybenzaldehyde. Stirring at 100 °C for 2 h. Trituration with 16 mL hot toluene yielded the pure product. Yield: 110.0 mg (66%), brown solid. *R_f_* (MeOH): 0.55. ^1^H NMR (300 MHz, DMSO-*d*_6_): *δ* (ppm) = 11.65 (s, 1H, OH), 9.69 (br s, 1H, OH), 7.82 (s, 1H, CH=N), 7.11 (d, ^3^*J* = 8.4 Hz, 1H, Ar-H), 6.28 (dd, ^3^*J* = 8.4 Hz, ^4^*J* = 2.1 Hz, 1H, Ar-H), 6.21 (d, ^4^*J* = 2.1 Hz, 1H, Ar-H), 2.99 (br s, 4H, 2CH_2_), 2.65 (br s, 4H, 2CH_2_), 2.30–2.27 (m, 1H, CH), 1.75–1.73 (m, 4H, 2CH_2_), 1.59–1.56 (m, 1H, CH), 1.19–1.05 (m, 5H, 2CH_2_, CH). ^13^C NMR (75.5 MHz, DMSO-*d*_6_): *δ* (ppm) = 158.8 (Cq-OH), 158.5 (Cq-OH), 141.1 (CH=N), 130.6 (CHAr), 111.5 (Cq), 106.9 (CHAr), 102.3 (CHAr), 62.2 (CH), 51.6 (2CH_2_), 47.4 (2CH_2_), 28.3 (2CH_2_), 25.8 (CH_2_), 25.2 (2CH_2_). Mp: 247–249 °C (dec.). HRMS (EI^+^): *m*/*z*: calcd for C_17_H_25_O_2_N_3_ [M]^+^: 303.1947; found 303.1942.

#### 4.2.46 (*E*)-4-((4-Cyclopentylpiperazin-1-ylimino)methyl)benzene-1,3-diol (**3g**)

According to GP-1: 150 mg (887 μmol, 1.00 equiv) 4-cyclopentylpiperazine-1-amine, 2.0 mL toluene, 123 mg (887 μmol, 1.00 equiv) 2,4-dihydroxybenzaldehyde. Stirring at 100 °C for 2 h. Trituration with 16 mL hot toluene yielded the pure product. yield: 186.1 mg (76%), beige solid. *R_f_* (MeOH): 0.57. ^1^H NMR (300 MHz, DMSO-*d*_6_): *δ* (ppm) = 11.63 (s, 1H, OH), 9.69 (br s, 1H, OH), 7.82 (s, 1H, CH=N), 7.12 (d, ^3^*J* = 8.4 Hz, 1H, Ar-H), 6.28 (dd, ^3^*J* = 8.4 Hz, ^4^*J* = 2.1 Hz, 1H, Ar-H), 6.22 (d, ^4^*J* = 2.1 Hz, 1H, Ar-H), 3.00 (br s, 4H, 2CH_2_), 2.58–2.49 (m, 4H, 2CH_2_), 2.46–2.44 (m, 1H, CH), 1.81–1.78 (m, 2H, CH_2_), 1.63–1.47 (m, 4H, 2CH_2_), 1.36–1.30 (m, 2H, CH_2_). ^13^C NMR (75.5 MHz, DMSO-*d*_6_): *δ* (ppm) = 158.8 (Cq-OH), 158.5 (Cq-OH), 141.2 (CH=N), 130.6 (CHAr), 111.5 (Cq), 106.9 (CHAr), 102.3 (CHAr), 66.3 (CH), 51.2 (2CH_2_), 50.6 (2CH_2_), 29.9 (2CH_2_), 23.6 (2CH_2_). Mp: 237–239 °C (dec.). HRMS (EI^+^): *m*/*z*: calcd for C_16_H_23_O_2_N_3_ [M]^+^: 289.1790; found 289.1795.

#### 4.2.47 (*E*)-4-((4-Phenylpiperidine-1-ylimino)methyl)benzene-1,3-diol (**3h**)

According to GP-1: 50.0 mg (284 μmol, 1.00 equiv) 4-phenylpiperidine-1-amine (30), 1.0 mL toluene, 39.0 mg (284 μmol, 1.00 equiv) 2,4-dihydroxybenzaldehyde. Stirring at 100 °C for 2.5 h. Final purification by recrystallization from 2.0 mL toluene yielded the pure product. Yield: 48.0 mg (57%), yellow crystals. ^1^H NMR (300 MHz, CDCl_3_): *δ* (ppm) = 11.77 (s, 1H, OH), 9.66 (br s, 1H, OH), 7.89 (s, 1H, CH=N), 7.34–7.27 (m, 4H, Ar-H), 7.23–7.20 (m, 1H, Ar-H), 7.14 (d, ^3^*J* = 8.4 Hz; 1H, Ar-H), 6.29 (dd, ^3^*J* = 8.4 Hz, ^4^*J* = 2.4 Hz, 1H, Ar-H), 6.23 (d, ^4^*J* = 2.4 Hz, 1H, Ar-H) 3.74–3.70 (m, 2H, CH_2_), 2.67–2.59 (m, 3H, CH, CH_2_), 1.88–1.80 (m, 4H, 2CH_2_). ^13^C NMR (75.5 MHz, CDCl_3_): *δ* (ppm) = 158.7 (Cq-OH), 158.5 (Cq-OH), 145.5 (Cq), 141.1 (CH=N), 130.6 (CHAr), 128.3 (2CHAr), 126.6 (2CHAr), 126.1 (CHAr), 111.7 (Cq), 106.8 (CHAr), 102.3 (CHAr), 51.6 (2CH_2_), 41.0 (CH), 31.7 (2CH_2_). Mp: 139–142 °C. HRMS (EI^+^): *m*/*z*: calcd for C_18_H_20_O_2_N_2_ [M]^+^: 296.1525; found 296.1530.

#### 4.2.48 (*E*)-4-((4-benzylpiperidine-1-ylimino)methyl)benzene-1,3-diol (**3i**)

According to GP-1: 86.0 mg (453 μmol, 1.00 equiv, 96% purity) 4-benzylpiperidine-1-amine (34), 2.0 mL toluene, 60.1 mg (435 μmol, 1.0 equiv) 2,4-dihydroxybenzaldehyde. Stirring at 100 °C overnight. Yield: 134.0 mg (95%), yellow-brown solid. ^1^H NMR (300 MHz, MeOD): *δ* (ppm) = 7.82 (s, 1H, CH=N), 7.29–7.24 (m, 2H, Ar-H), 7.19–7.16 (m, 3H, Ar-H), 7.04 (d, ^3^*J* = 8.4 Hz, 1H, Ar-H), 6.30 (dd, ^3^*J* = 8.4 Hz, 4 J = 2.4 Hz, 1H, Ar-H), 6.25 (d, ^4^*J* = 2.4 Hz, 1H, Ar-H), 3.61–3.57 (m, 2H, CH_2_), 2.58 (d, ^3^*J* = 6.9 Hz, 2H, CH_2_), 2.54–2.46 (m, 2H, CH_2_), 1.79–1.75 (m, 2H, CH_2_), 1.71–1.63 (m, 1H, CH), 1.50–1.41 (m, 2H, CH_2_). ^13^C NMR (75.5 MHz, MeOD): *δ* (ppm) = 160.6 (Cq-OH), 160.5 (Cq-OH), 144.5 (CH=N), 141.6 (Cq), 132.3 (CHAr), 130.2 (2CHAr), 129.3 (2CHAr), 127.0 (CHAr), 113.4 (Cq), 108.1 (CHAr), 103.7 (CHAr), 53.3 (2CH_2_), 43.7 (CH_2_), 38.8 (CH), 32.1 (2CH_2_). Mp: 138–141 °C. HRMS (EI^+^): *m*/*z*: calcd for C_19_H_22_O_2_N_2_ [M]^+^: 310.1681; found 310.1683.

#### 4.2.49 (*E*)-4-((4-Phenyl-1,4-diazepan-1-ylimino)methyl)benzene-1,3-diol (**3j**)

According to GP-1: 31.7 mg (166 μmol, 1.00 equiv) 4-phenyl-1,4-diazepane-1-amine (40), 1.0 mL toluene, 21.9 mg (166 μmol, 1.00 equiv) 2,4-dihydroxybenzaldehyde. Stirring at 100 °C overnight. Yield: 50.0 mg (97%), brown-red oil. *R_f_* (CH/EtOAc 3:1): 0.75. ^1^H NMR (300 MHz, DMSO-*d*_6_): *δ* (ppm) = 11.39 (s, 1H, CH), 9.51 (br s, 1H, OH), 7.54 (s, 1H, OH), 7.18–7.12 (m, 2H, Ar-H), 7.06 (d, ^3^*J* = 8.4 Hz, 1 H, Ar-H), 6.75 (d, ^3^*J* = 8.4 Hz, 1 H, Ar-H), 6.58 (t, ^3^*J* = 7.2 Hz, 1H, Ar-H), 6.25 (dd, ^3^*J* = 8.4 Hz, ^4^*J* = 2.1 Hz, 1H, Ar-H), 6.19 (d, ^4^*J* = 2.1 Hz, 1H, Ar-H), 3.63–3.62 (m, 2H, CH_2_), 3.52–3.50 (m, 2H, CH_2_), 3.43–3.37 (m, 4H, 2CH_2_), 1.99–1.95 (m, 2H, CH_2_). ^13^C NMR (75.5 MHz, DMSO-*d*_6_): *δ* (ppm) =  = 157.8 (Cq-OH), 157.5 (Cq-OH), 147.1 (CH=N), 135.5 (Cq), 129.4 (CHAr), 129.2 (2CHAr), 115.5 (CHAr), 112.5 (Cq), 111.4 (2CHAr), 106.7 (CHAr), 102.3 (CHAr), 51.7 (CH_2_), 49.6 (CH_2_), 47.3 (CH_2_), 47.2 (CH_2_), 23.2 (CH_2_). HRMS (EI^+^): *m*/*z*: calcd for C_18_H_21_O_2_N_3_ [M]^+^: 311.1634; found 311.1616.

#### 4.2.50 4-((4-Phenylpiperazin-1-yl)methyl)benzene-1,2-diol (**8**)

A 25 mL Schlenk tube was flushed with argon and charged with 250 mg (1.81 mmol, 1.00 equiv) 3,4-dihydroxybenzaldehyde, 7.0 mL anhydrous DCE and 294 mg (0.28 mL, 1.81 mmol, 1.00 equiv) 1-phenylpiperazine. To the orange solution were added 538 mg (2.54 mmol, 1.40 equiv) sodium triacetoxyborhydride and 100 μL (1.81 mmol, 1.00 equiv) acetic acid. The colorless suspension was stirred at rt overnight, during which the color of the suspension turned bright yellow. The mixture was hydrolyzed by addition of saturated NaHCO_3_ solution (10 mL) and concentrated under reduced pressure. The residue was dissolved in EtOAc (30 mL) and water (30 mL) and the layers were separated. The aqueous layer was extracted with EtOAc (2 × 20 mL) and the combined organic layers were washed with brine (25 mL), dried over MgSO_4_ and concentrated under reduced pressure. Final purification by silica gel filtration (DCM/MeOH 19:1, *R_f_* = 0.38) yielded the pure product. Yield: 135.9 mg (26%), brown solid. ^1^H NMR (300 MHz, DMSO-*d*_6_): *δ* (ppm) = 8.87 (br s, 2H, 2OH), 7.19 (t, ^3^*J* = 7.8 Hz, 2H, Ar-H), 6.90 (d, ^3^*J* = 8.1 Hz, 2H, Ar-H), 6.78–6.73 (m, 2H, Ar-H), 6.68–6.65 (m, 1H, Ar-H), 6.56–6.53 (m, 1H, Ar-H), 3.32 (s, 2H, CH_2_), 3.10 (br s, 4H, 2CH_2_), 2.47–2.46 (m, 4H, 2CH_2_). ^13^C NMR (75.5 MHz, DMSO-*d*_6_): *δ* (ppm) = 150.9 (Cq), 144.9 (Cq-OH), 144.1 (Cq-OH), 128.8 (2CHAr), 128.6 (Cq), 119.7 (CHAr), 118.6 (CHAr), 116.2 (CHAr), 115.2 (2CHAr), 115.0 (CHAr), 61.8 (CH_2_), 52.4 (2CH_2_), 48.1 (2CH_2_). Mp: 100–102 °C. HRMS (EI^+^): *m*/*z*: calcd for C_17_H_20_O_2_N_2_ [M]^+^: 284.1525; found 284.1540.

#### 4.2.51 3,4-Dimethoxy-*N*-(4-phenylpiperazin-1-yl)benzamide (**10**)

A 25 mL Schlenk tube was charged with 100 mg (549 μmol, 1.00 equiv) 3,4-dimethoxybenzoic acid, 218 mg (140 μL, 1.83 mmol, 3.33 equiv) thionylchloride and one catalytic drop of anhydrous DMF. The light yellow suspension was heated under reflux for 3.5 h. The excess thionylchloride was removed under high vacuum over a cooling trap to obtain a light yellow solid (acid chloride). A 25 mL one-neck round bottom flask was charged with 117 mg (659 μmol, 1.20 equiv) 4-phenylpiperazin-1-amine and 2.0 mL 10% aqueous NaOH (2.0 mL). The solution was cooled to 0 °C and a solution of the acid chloride in 1 mL absolute DCM was added. The mixture was stirred at rt for 30 min, during which a colorless solid precipitated. The solid was collected by filtration, washed with DCM and dried under vacuum. Final purification by recrystallization from EtOH (25 mL) yielded the pure product. Yield: 98.0 mg (53%), colorless solid. ^1^H NMR (300 MHz, CDCl_3_): *δ* (ppm) = 7.40 (br s, 1H, NH), 7.31–7.27 (m, 3H, Ar-H), 6.98–6.84 (m, 5H, Ar-H), 3.93–3.92 (m, 6H, 2OCH_3_), 3.40 (m, 4H, 2CH_2_), 3.12 (m, 4H, 2CH_2_). ^13^C NMR (75.5 MHz, CDCl_3_): *δ* (ppm) = 165.2 (C=O), 151.9 (Cq-OCH_3_), 150.9 (Cq-OCH_3_), 149.1 (Cq), 129.2 (2CHAr), 126.2 (Cq), 120.3 (CHAr), 119.4 (CHAr), 116.5 (2CHAr), 110.8 (CHAr), 110.2 (CHAr), 56.0 (2CH_2_), 55.4 (2OCH_3_), 48.9 (2CH_2_).

#### 4.2.52 *N*-(3,4-Dimethoxyphenyl)-4-phenylpiperazin-1-carboxamide (**12**)

A 25 mL Schlenk tube was dried under vacuum, filled with argon and charged with 500 mg (3.26 mmol, 1.00 equiv) 3,4-dimethoxyaniline and 6.1 mL absolute EtOH. 1.07 g (4.90 mmol, 1.50 equiv) di-*tert*-butyl dicarbonate were added slowly and the suspension was stirred at rt for 2.5 h. The mixture was transferred to a one-neck round bottom flask and concentrated under reduced pressure. Drying under high vacuum overnight yielded the product which was used in the next reaction without further purification. A 15 mL Schlenk tube was dried under vacuum, filled with argon and charged with 176 mg (166 μL, 1.09 mmol, 1.10 equiv) 1-phenylpiperazine and 2.0 mL absolute THF. The yellow solution was cooled to 0 °C and 76.0 mg (474 μL, 1.18 mmol, 1.20 equiv) *n*-butyllithium were added. The mixture was warmed to rt and a solution of 250 mg (987 μmol, 1.00 equiv) of previously prepared *tert*-butyl-3,4-dimethoxyphenylcarbamate in 2.0 mL absolute THF was added dropwise. The greenbrown solution was heated under reflux for 5 h. The mixture was hydrolyzed with 5 mL 5% aqueous HCl (5 mL). The aqueous layer was extracted with DCM (3 × 5 mL) and the combined organic layers were washed with 5 mL saturated aqueous NaHCO_3_ (5 mL), water (5 mL) and brine (5 mL). The organic layer was dried over Na_2_SO_4_ and concentrated under reduced pressure. Final purification by column chromatography (CH/EtOAc, size: 18.5 × 2.5 cm, 30 g SiO_2_) yielded the pure product. Yield: 81.0 mg (25%), yellow-orange solid. *R_f_* (CH/EtOAc 1:1): 0.19. ^1^H NMR (300 MHz, DMSO-*d*_6_): *δ* (ppm) = 8.44 (br, 1H, NH), 7.26–7.17 (m, 3H, Ar-H), 7.00–6.97 (m, 3H, Ar-H), 6.84–6.79 (m, 2H, Ar-H), 3.71 (s, 3H, OCH_3_), 3.70 (s, 3H, OCH_3_), 3.59–3.56 (m, 4H, 2CH_2_), 3.17–3.13 (m, 4H, 2CH_2_). ^13^C NMR (75.5 MHz, DMSO-*d*_6_): *δ* (ppm) = 155.0 (C=O), 150.8 (Cq-OCH_3_), 148.3 (Cq-OCH_3_), 143.9 (Cq), 133.9 (Cq), 128.8 (2CHAr), 119.1 (CHAr), 115.7 (2CHAr), 112.0 (CHAr), 111.5 (CHAr), 105.3 (CHAr), 55.7 (OCH_3_), 55.2 (OCH_3_), 48.3 (2CH_2_), 43.5 (2CH_2_). Mp: 122 °C–125 °C.

#### 4.2.53 3,4-Difluorophenyl-4-phenylpiperazin-1-carboxylate (**14**)

A 15 mL Schlenk tube was dried under vacuum, filled with argon and charged with 500 mg (3.84 mmol, 1.00 equiv) 3,4-difluorophenol, 2.0 mL absolute DCM and 624 mg (3.84 mmol, 1.00 equiv) 1,1′-carbonyldiimidazole. The yellow mixture was stirred at rt for 1.5 h. To reach full conversion of the starting material (GC–MS analysis) 605 mg (3.72 mmol, 0.97 equiv) 1,1′-carbonyldiimidazol, dissolved in 1 mL DCM, were added and the mixture was stirred at rt for further 20 h. The product solution was used in the next reaction without work up or any purification step. 623 mg (590 μL, 3.84 mmol, 1.00 equiv) 1-phenylpiperazine were added to the product mixture of 3,4-difluorophenyl-1*H*-imidazol-1-carboxylate. After 1 h stirring at rt once more 25.0 mg (20 μL, 150 μmol, 0.04 equiv) 1-phenylpiperazine were added to reach full conversion (GC–MS analysis). The mixture was stirred at rt for 2 h and washed with water (2 × 5 mL). The organic layer was dried over MgSO_4_ and concentrated under reduced pressure. As the obtained solid was not pure enough, even after silica gel filtration (CH/EtOAc 5:1, *R_f_* = 0.18), it was dissolved in DCM (5 mL) and washed with 5 mL 2 M NaOH (5 mL). The organic layer was dried over Na_2_SO_4_, concentrated under reduced pressure and dried under high vacuum to yield the pure product. Yield: 246.0 mg (20%), light yellow solid. *R_f_* (DCM): 0.49. ^1^H NMR (300 MHz, CDCl_3_): *δ* (ppm) = 7.34–7.29 (m, 2H, Ar-H), 7.15 (q, ^3^*J* = 9.0 Hz, Ar-H), 7.07–6.86 (m, 5H, Ar-H), 3.82–3.75 (m, 4H, 2CH_2_), 3.25 (t, ^3^*J* = 5.4 Hz, 4H, 2CH_2_). ^13^C NMR (75.5 MHz, CDCl_3_): *δ* (ppm) = 153.0 (C=O), 151.7, 151.5, 148.4, 148.2 (^1^*J*_C–F_ = 249.5 Hz, ^2^*J*_C–F_ = 13.9 Hz, Cq-F), 149.7, 149.6, 146.5, 146.3 (^1^*J*_C–F_ = 245.7 Hz, ^2^*J*_C–F_ = 12.5 Hz, Cq-F), 147.0, 146.9, 146.8, (^3^*J*_C–F_ = 8.8 Hz, ^4^*J*_C–F_ = 3.2 Hz, Cq), 129.3 (2CHAr), 120.9 (CHAr), 117.7, 117.6, 117.5 (CHAr), 117.2 (Cq), 116.9 (2CHAr), 111.9, 111.6 (^2^*J*_C–F_ = 19.9 Hz, CHAr), 49.6 (2CH_2_), 43.9 (2CH_2_). Mp: 94–96 °C. HRMS (EI^+^): *m*/*z*: calcd for C_17_H_16_O_2_N_2_F_2_ [M]^+^: 318.1180; found 318.1182.

### 4.3 Triglyceride hydrolase activity assay

For the determination of TG hydrolase activity cell lysates of COS-7 cells overexpressing recombinant murine ATGL were used. Therefore, monkey embryonic kidney cells (Cos-7; ATCC CRL-1651) were cultivated in Dulbeccos modified eagles medium (DMEM, GIBCO, Invitrogen Corp., Carlsbad, CA), containing 10% fetal calf serum (FCS, Sigma–Aldrich) and antibiotics (100 IU/mL penicillin and 100 μg/mL streptomycin) at standard conditions (37 °C, 5% CO_2_, 95% humidified atmosphere). Cells were transfected with 1 μg DNA complexed to Metafectene (Biontex GmbH, Munich, Germany) in serum free DMEM. After 4 h the medium was replaced by DMEM supplemented with 10% FCS. For the preparation of cell lysates, cells were washed with 1× phosphate buffered saline, collected using a cell scraper, and disrupted in buffer A (0.25 M sucrose, 1 mM EDTA, 1 mM dithiothreitol, 20 μg/mL leupeptine, 2 μg/mL antipain, 1 μg/mL pepstatin, pH 7.0) by sonication (Virsonic 475, Virtis, Gardiner, NJ). Nuclei and unbroken cells were removed by centrifugation (1000×*g*, 4 °C, for 10 min). Protein concentration of the cell lysates were determined by Bio-Rad protein assay according to the manufacturer’s protocol (Bio-Rad 785, Bio-Rad Laboratories, Munich, Germany), using BSA as standard.

The triolein (TO) substrate was prepared as described. ^20^ In brief, 330 μM triolein (40,000 cpm/nmol) (GE Healthcare) was emulsified with 45 μM phosphatidylcholine/phosphatidylinositol (3:1) (Sigma) in 100 mM potassium phosphate buffer (pH 7.0) by sonication (Virsonic 475, Virtis) and was adjusted to 5% (w/v) fatty acid free bovine serum albumin (BSA, Sigma). 100 μL (20 μg protein) cell lysate or 2 μl purified recombinant LPL (Sigma–Aldrich) were incubated with 100 μL TO substrate in the presence or in the absence of inhibitors in a water bath for 1 h at 37 °C. After incubation, the reaction was terminated by adding 3.25 mL of methanol/chloroform/heptane (10:9:7) and 1 mL of 0.1 M potassium carbonate, 0.1 M boric acid (pH 10.5). After centrifugation (800 g, 15 min), the radioactivity in 1 mL of the upper phase was determined by liquid scintillation counting.

## Supplementary data

Supplementary data Selectivity tests of compounds **1a** and **1h** and toxicity test of **1h**.
