# Supplementary material for: Structure–activity studies in the development of a hydrazone based inhibitor of adipose-triglyceride lipase (ATGL)
Source: Bioorg Med Chem. 2015 Jun 15;23(12):2904–16. doi: 10.1016/j.bmc.2015.02.051 (PMC4457358; doi:10.1016/j.bmc.2015.02.051)
Supplement: Supplementary data — Selectivity tests of compounds 1a and 1h and toxicity test of 1h. [file mmc1.pdf]

Supplementary Material

Structure-activity studies in the development of a hydrazone based inhibitor of adipose-triglyceride lipase (ATGL)

Nicole Mayer, Martina Schweiger, Michaela-Christina Melcher, Christian Fledelius, Rudolf Zechner, Robert Zimmermann and Rolf Breinbauer

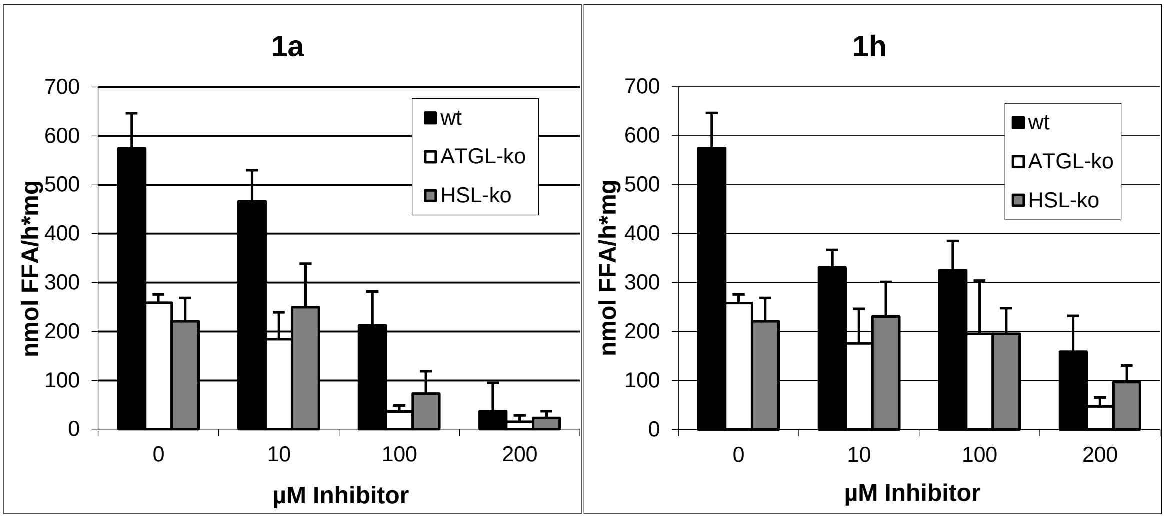

Fig. S1: Selectivity tests of **1a** and **1h** with white adipose tissue fat pads of wt, ATGL-ko, and HSL-ko mice

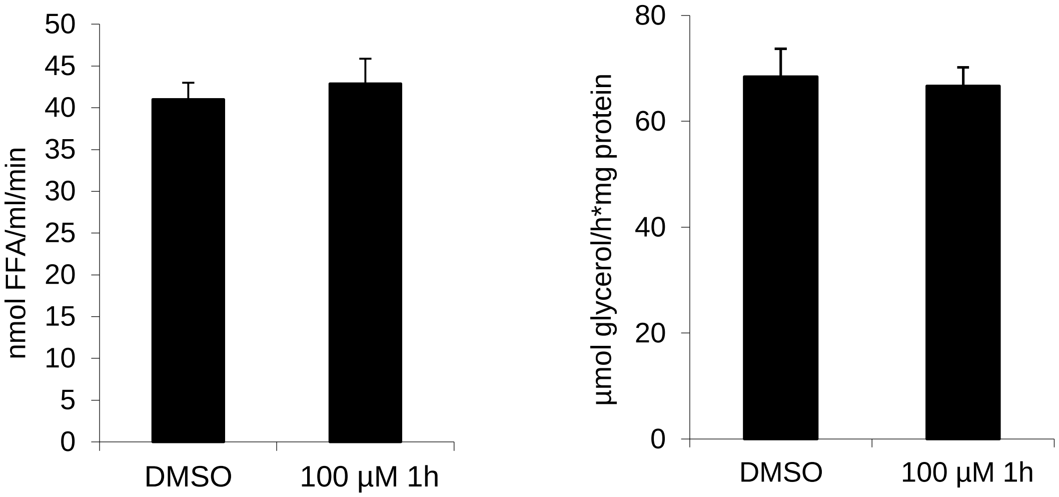

Fig S2. LPL (left) and Monoglyceride hydrolase (MGH) (right) activities in the absence or presence of 100 µM compound **1h**.

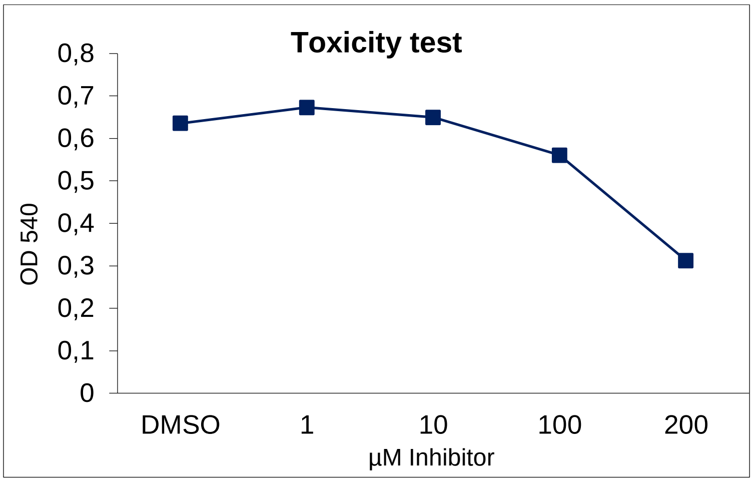

Fig. S3: Toxicity test of inhibitor **1h** using Tox 4-test with AML-12 mouse hepatocytes cells

Lipolysis of isolated WAT organ cultures - Gonadal fat pads of wild-type, ATGL deficient (ATGL-ko), and HSL deficient (HSL-ko) mice were surgically removed and washed several times with PBS. Tissue pieces (~15 mg) were preincubated in DMEM containing 0, 10, 100, and 200 µM inhibitors for 8 h at C37°C, 5% $CO_{2,}$ 95 % humidified atmosphere. Thereafter, the medium was replaced by DMEM containing 2 % BSA (fatty acid-free) and 0, 10, 100, and 200 µM inhibitors, and incubated for another 60 min at 37°C. Then, aliquots of the medium were removed and analyzed for FA and glycerol content using commercial kits (HR Series NEFA-HR(2), WAKO Diagnostics, Neuss, Germany; Sigma, St. Louis, MO). For protein determinations, fat pads were washed extensively with PBS and lysed in 0.3 N NaOH/0.1 % SDS. Protein measurements were performed using the BCA reagent (Pierce Rockfort, IL).

Toxicity test for inhibitors in AML-12 mouse hepatocytes. For Tox-4 (Sigma-Aldrich) *in vitro* viability assays, cells were seeded at an initial density of $1.5x10^{5}$ cells per well in 12-well plates and cultured under standard conditions for 24 hours. The next day, cells were pretreated with different concentrations of inhibitors dissolved in DMSO or DMSO as control for two hours. Medium was replaced by an identical fresh medium containing 1/10 volume of neutral red solution and incubated again for 2 hours. Thereafter cells were extensively washed with PBS. After solubilization for 10 min at room temperature absorbance was measured at 540 nm.

Determination of MG hydrolase activity. Monoacylglycerol hydrolase activities were determined using recombinant, purified mMGL and *rac*-1-(3)-oleoylglycerol as substrate as described （Taschler, U. et al. Monoglyceride lipase deficiency in mice impairs lipolysis and attenuates diet-induced insulin resistance. J Biol Chem 286, 17467-77 (2011)).
